# The massive 2016 marine heatwave in the Southwest Pacific: An “El Niño–Madden-Julian Oscillation” compound event

**DOI:** 10.1126/sciadv.adp2948

**Published:** 2024-10-09

**Authors:** Cyril Dutheil, Shilpa Lal, Matthieu Lengaigne, Sophie Cravatte, Christophe Menkès, Aurore Receveur, Florian Börgel, Matthias Gröger, Fanny Houlbreque, Romain Le Gendre, Inès Mangolte, Alexandre Peltier, H. E. Markus Meier

**Affiliations:** ^1^ Department of Physical Oceanography and Instrumentation, Leibniz Institute for Baltic Sea Research Warnemünde, Rostock, Germany.; ^2^ MARBEC, University of Montpellier, CNRS, IFREMER, IRD, Sète, France.; ^3^ Université de Toulouse, LEGOS (CNES/CNRS/IRD/UT3), Nouméa, Nouvelle-Calédonie, France.; ^4^ ENTROPIE, IRD, Univ. de la Nouvelle Calédonie, Univ. de la Réunion, CNRS, Ifremer, Nouméa, Nouvelle-Calédonie, France.; ^5^ CESAB-FRB, 5 Rue de l’École de Médecine, 34000 Montpellier, France.; ^6^ Météo France, Nouméa, Nouvelle Calédonie, France.

## Abstract

El Niño typically induces cooling in the Southwest Pacific Ocean during austral summers, usually leading to decreased marine heatwave frequency and severity. However, the 2016 extreme El Niño unexpectedly coincided with the longest and most extensive marine heatwave ever recorded in the region. This heatwave, spanning over 1.7 million square kilometers, persisting for 24 days with a peak intensity of 1.5°C, resulted in massive coral bleaching and fish mortality. This exceptional warming resulted from anomalously strong shortwave radiation and reduced heat loss via latent heat fluxes, owing to low wind speed and increased air humidity. These anomalies are attributed to a rare combined event “Madden-Julian Oscillation and extreme El Niño.” Following 10 February, the rapid dissipation of this marine heatwave results from the most intense cyclone ever recorded in the South Pacific. The hazardous ecological impacts of this extreme event highlight the needs for improving our understanding of marine heatwave–driving mechanisms that may result in better seasonal predictions.

## Teaser

Even during El Niño, massive marine heatwaves can occur in the Southwest Pacific when combined with the Madden-Julian Oscillation.

## INTRODUCTION

**Fig. 1.**
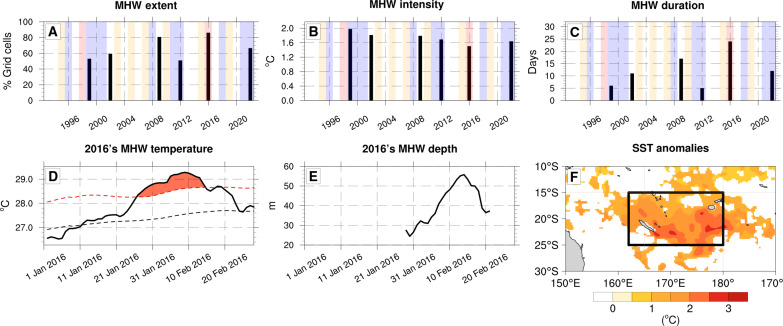
Austral summer (December-January-February) marine heatwave (MHW) statistics from 1993 to 2022. (**A**) The maximum proportion of our study area experiencing MHW conditions (in percentages of grid cells), (**B**) maximum intensity (in degrees Celsius), and (**C**) the duration of MHWs (in days). A MHW is detected when the event covers 50% of our study region during at least 5 days. How the three statistics are calculated is explained in Materials and Methods. Vertical color shadings on these panels indicate El Niño–Southern Oscillation phases, moderate El Niño (yellow), La Niña (blue), and extreme El Niño (red). (**D**) The SST time series and (**E**) vertical extent (in meters) of the 2016 MHW averaged over the study region [black box in (F)] from 1 January to 28 February 2016. Black dashed line on (D) is the temperature climatology, and the red dashed line is the 90th percentile of SST (threshold for the MHW detection). (**F**) SST anomalies on 6 February 2016, day when the largest proportion of the region experienced MHW conditions. Regions where there is no MHW are masked in white. The black box shows the study area (162°E to 180°E; 15°S to 25°S) used for the statistics and the temperature budget calculation. (A) to (D) and (F) were performed with National Oceanic and Atmospheric Administration (NOAA) Optimum Interpolation (OI) SST V2 High Resolution Dataset and (E) comes from GLORYS reanalysis.

Marine heatwaves (MHWs) are the most prominent ocean temperature extremes (*1*). Depending on their intensity, duration, or extension, they have the potential to inflict severe damage on marine ecosystems, including mass mortalities, species migration, and massive coral bleaching events (*1*–*4*). Considering a fixed baseline, frequency and intensity of the MHWs are expected to increase under climate change (*5*, *6*); thus, it is crucial to understand their underlying mechanisms, especially within marine biodiversity hotspots like the Melanesian islands in the Southwest Pacific (*7*). During the 2016 austral summer, an extreme El Niño event coincided with a massive MHW in the Southwest Pacific (Fig. 1). This MHW triggered an unprecedented coral bleaching event on the New Caledonia reef (*8*, *9*), affecting 87% of the sampled coral population (292 monitored sites). Fortunately, the abrupt dissipation of this MHW facilitated the recovery of 70% of the corals. In addition, this MHW inflicted coral bleaching and massive fish mortalities on other Melanesian islands, including Fiji, Vanuatu, and Kiribati (*10*, *11*). Although both central and eastern Pacific El Niño events are typically associated with cooler surface temperatures in this region (*12*), substantial ecological impacts were observed during the 2016 extreme El Niño event, a mix between central and eastern El Niño (*13*, *14*) (figs. S1 and S2). This underlines the necessity of unraveling the underlying mechanisms leading to the growth and demise of this exceptional event to prevent misinterpretations that can lead to forecasting errors in the future.

MHWs can be triggered by a variety of atmospheric and oceanic processes (*15*). Although there is a wealth of literature on the mechanisms behind extreme El Niño warmings (*13*, *16*, *17*), there remains a gap in our understanding of shorter MHWs, such as those lasting a few weeks. In the Southwest Pacific, El Niño typically brings colder and drier conditions (*12*, *18*), reducing the risk of MHWs (*15*, *19*), contrasting with the warming and moistening observed in the central and eastern Equatorial Pacific regions, where the risk of MHWs is higher. Consequently, La Niña events have been identified as the main climate mode at the origin of MHWs in the Southwest Pacific (*15*, *19*). At intraseasonal timescales, specific phases of the Madden Julian Oscillation (MJO) (*20*) suppress atmospheric convection, increase solar radiation, and reduce surface wind speed in the South Pacific, thus having the potential to warm surface waters and increase the likelihood of MHWs. In addition, tropical cyclones, operating at even shorter temporal timescales, can interact with lower-frequency variability, further shaping the dynamics of MHWs (*21*).

In this study, we used oceanic simulations together with an upper-ocean temperature budget to elucidate the primary drivers behind this extensive MHW. Contrary to direct El Niño influence, our findings suggest that this event was predominantly triggered by the passage of a suppressed phase of a strong MJO originating in the Indian Ocean. This led to notable positive anomalies in shortwave radiation and reduced heat loss from the upper ocean via latent heat fluxes, facilitated by weaker-than-normal winds and increased air humidity. Furthermore, we show how El Niño indirectly enhanced this MJO effect by displacing the South Pacific Convergence Zone (SPCZ) (*22*) equatorward—a well-known effect of El Niño in that region, thereby amplifying the MJO-induced anomalies. The extraordinary fast decline of this MHW likely prevented more intense long-term biological effects, facilitating for instance the recovery of coral reefs. We related this decline to the passage of Tropical Cyclone Winston, one of the most intense ever recorded in the South Pacific, crossing the region within 2 weeks.

## RESULTS

### Description of the 2016 MHW event

To detect MHWs, we used the method outlined by Hobday *et al.* (*1*), using the seasonally varying 90th percentile of the daily sea surface temperature (SST). As discussed by Amaya *et al.* (*23*), this method is dependent on the baseline chosen. Here, we used a fixed baseline for the 1993–2022 period, which is well suited for representing the biological impacts of MHWs as the thermal tolerance of many species such as corals is threshold based (*24*). Subsequently, we excluded events covering less than 50% of the study area within the targeted region [black box in Fig. 1F; (162°E to 180°E; 15°S to 25°S)], to select the most extensive events. Figure 1 illustrates the identification of six spatially extensive MHWs occurring during austral summer. Notably, the 2016 event stands out with a coverage extending up to 90% of the total area (1.7 Mkm^2^), an average intensity of 1.5°C, and a duration of 24 days, making it the most extensive and longest event within this time frame. In addition, it displays the highest cumulative intensity, due to the combined effect of its intensity and duration (not shown). We assessed our results with larger size domains, and the 2016 MHW remains the most extensive and longest in most cases (fig. S3). Furthermore, it is the only extensive MHW in this region that has occurred during El Niño contrasting with three of six MHWs occurring during a La Niña event. During the 2016 austral summer, SST in this region exhibited a gradual increase from 1 to 20 January, exceeding the climatological average on 10 January (black dashed line in Fig. 1D). Subsequently, the warming accelerates, exceeding the 90th percentile of SST distribution on 23 January, hence qualifying the event as a MHW, which fades from 15 February onwards. The vertical extent of this event ranges from 25 to 60 m, i.e., two to four times deeper than the mixed-layer depth (fig. S4).

### Physical processes

To identify the main drivers of this MHW, we conducted a temperature budget for the 0- to 20-m layer (see Eq. 1 in Materials and Methods; approximately the mixed-layer depth) using data from the Global Ocean Reanalysis and Simulations 12v1 product (GLORYS) (*25*). GLORYS assimilates multiple observational datasets, ensuring a robust representation of real-world conditions. The temperature budget is calculated over the GLORYS period, i.e., 1993–2019, followed by the calculation of a daily climatology using an 11-day window centered on each day of year, akin to the methodology used for MHWs detection. Subsequently, this budget is integrated over time from 1 January to 28 February.

#### Growth of the 2016 MHW

**Fig. 2.**
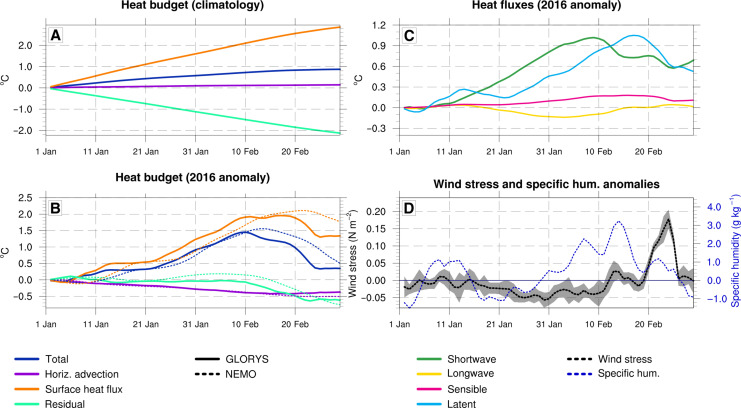
Upper (0- to 20-m) ocean temperature budget analyzed within the (162°E to 180°E; 25°S to 15°S) region (black box in Fig. 1F). (**A**) Time series for January-February depicting the time-integrated daily climatology of temperature budget terms (in degrees Celsius) over 1993–2019. (**B**) Time series of time-integrated anomalies of temperature budget terms from January 2016 to February 2016 (in degrees Celsius) relative to the daily climatology. Positive (negative) values indicate anomalously warming (cooling) contribution to the 20-m heat budget. In (A) and (B), blue line represents temperature, purple line is the contribution of horizontal advection, the orange line is the contribution of air-sea heat flux, and the green line is the residual. Solid lines indicate heat budget calculation from GLORYS, while the dashed lines depict results from a NEMO simulation with online mixed-layer temperature budget calculation. (**C**) Breakdown of the air-sea heat fluxes anomalies (in degrees Celsius) from January to February 2016 with respect to the daily climatology: shortwave radiation (green), net longwave radiation (orange), sensible heat flux (pink) and latent heat flux (blue). (**D**) Wind stress anomalies (in newtons per square meter; left vertical axis and black dashed line) and the specific humidity anomalies (in grams per kilogram; right vertical axis and blue dashed line). The gray envelop represents the time standard deviation of the wind stress in the study region.

Figure 2 (A and B) shows the time-integrated upper-ocean temperature budget for both climatological conditions and anomalies during the 2016 MHW. Under normal conditions, the climatological temperature increase in the 0- to 20-m layer primarily stems from increased surface heat fluxes, while the residual term tends to cool and horizontal advection contributes minimally. During the 2016 MHW, the sharp anomalous warming from 1 January to 10 February is predominantly driven by anomalous strong surface heat fluxes, while the horizontal advection has a weak cooling effect. The residual term has a negligible impact during this period. However, as GLORYS assimilates data, the heat budget closure is not ensured. A Nucleus for European Modelling of the Ocean (NEMO) simulation with an online exact calculation of the mixed-layer temperature budget, shown by the dashed versus solid lines in Fig. 2B, confirms GLORYS results derived offline from daily outputs with a fixed mixed-layer depth. We then identify the main contributors to ocean heat gain by examining the increased net surface heat fluxes. We separate shortwave and net longwave radiation, as well as latent and sensible heat fluxes, as shown in Fig. 2C (for detailed methodology, see Eq. 2 in Materials and Methods). This analysis reveals that the positive influx of shortwave radiation is the main driver, resulting in 1°C warming from 10 January to 10 February. In addition, the reduction in oceanic heat losses due to latent fluxes (lower evaporation) also initially contributes to the growth of the 2016 MHW, accounting for 0.3°C warming between 5 and 12 January, followed by an almost 1°C warming between 23 January and 17 February. This reduction in latent heat fluxes is attributed to the combined effect of abnormally weak winds followed by high moisture, which reduced cooling by evaporation (black and blue dashed curves in Fig. 2C). Last, we quantified the influence of a shallower-than-usual mixed-layer depth. Figure S5 shows the difference between the time-integrated mixed-layer temperature budget calculated on the actual mixed-layer depth and the climatological mixed-layer depth. The temperature difference is very small, highlighting the weak influence of a shallower mixed-layer depth on the 2016 MHW because its effect on shortwave radiation and latent heat fluxes is opposite. In addition, the cooling by vertical mixing is more efficient with a shallower mixed layer.

#### Decline of the 2016 MHW

The 2016 MHW reaches its peak around 10 February, after which surface temperature rapidly declines until 22 February. Initially, this temperature decrease is driven by intensified cooling from the residual term including the entrainment at the base of the mixed layer, the vertical advection, and the vertical mixing. The cooling is initially (around 31 January) due to the entrainment at the base of the mixed layer and then to the vertical mixing, after 10 February, in response to the positive wind stress anomalies (Fig. 2D and fig. S6). From mid-February, the anomalous Ekman pumping produces a cooling by vertical advection of temperature (fig. S6). Around 20 February, the temperature decline accelerates in response to a strong cooling through latent heat fluxes at a time when the wind speed abruptly increases (black dotted curve in Fig. 2D).

### Synoptic and mesoscale patterns

**Fig. 3.**
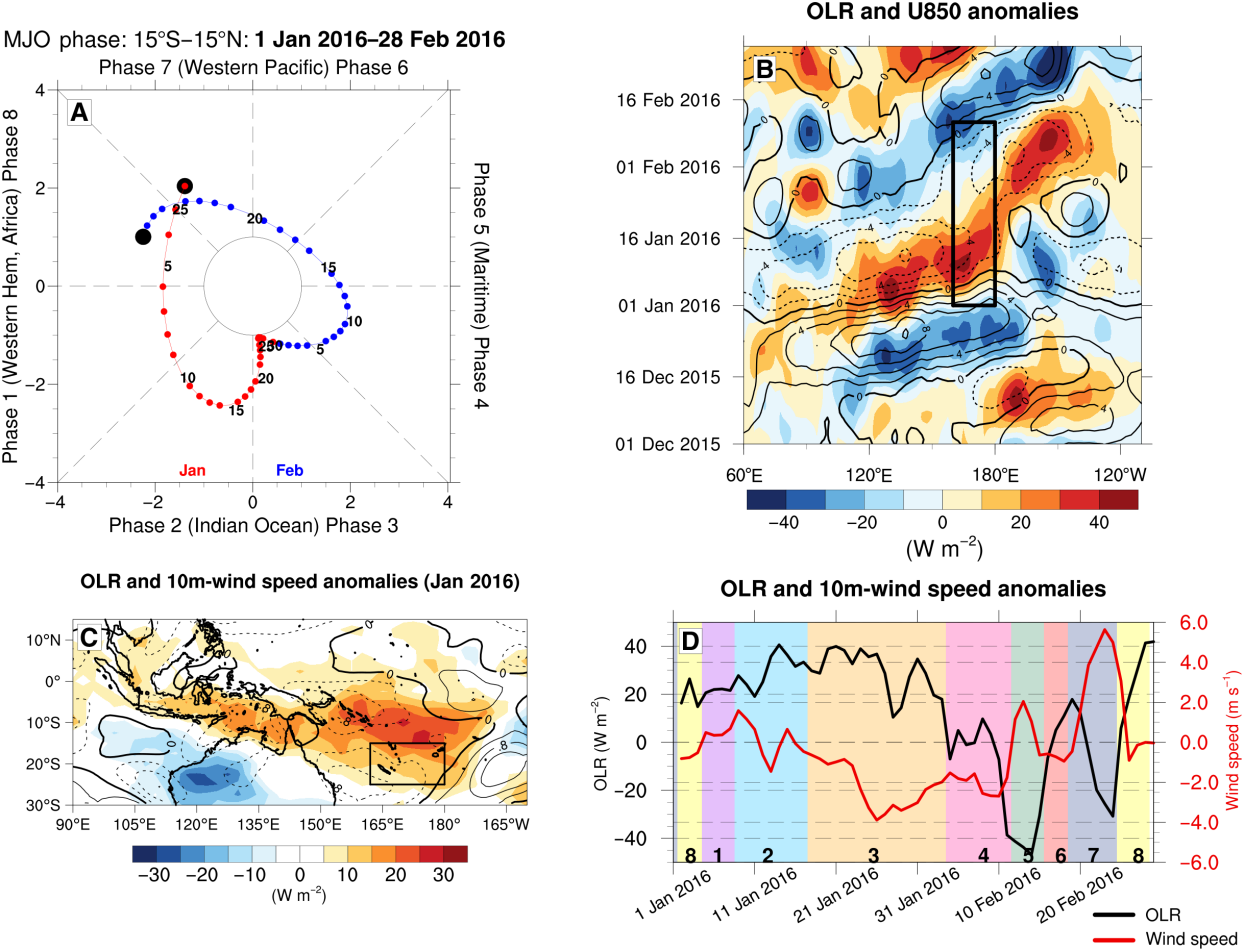
The 2016 austral summer Madden-Julian Oscillation (MJO) event. (**A**) Phase-space diagram of the MJO index showing daily phase (quadrant) and magnitude (distance from center) of the MJO from 1 January to 28 February 2016. January days are depicted in red, February days in blue, and the numbers indicating the days of the month. (**B**) Hovmöller diagram of intraseasonal outgoing longwave radiation (OLR; shading in watts per square meter) and zonal wind at 850-hPa (U850; contours lines with 2 m s^−1^ interval and negative values dashed) anomalies averaged between 20°S and 0°N for the austral summer (December-January-February) 2016. The black box indicates the period from 1 January to 10 February within the region (160°E to 180°E). (**C**) January 2016 OLR (in watts per square meter; shading) and surface wind speed anomalies (in meters per second; contours lines with 0.4 m s^−1^ interval and negative values dashed). Here, intraseasonal anomalies are extracted via a Lanczos band-pass filter to retain only frequencies between 20 and 100 days, thus highlighting MJO-related signature. The black box shows the study area (162°E to 180°E; 15°S to 25°S). (**D**) Domain average OLR (in watts per square meter; black line) and 10-m wind speed anomalies (in meters per second; red line) relative to daily climatologies. Vertical colors shadings indicate MJO phases. Wind and OLR data used in this figure were respectively extracted from ERA5 reanalysis and NOAA OLR daily data.

**Fig. 4.**
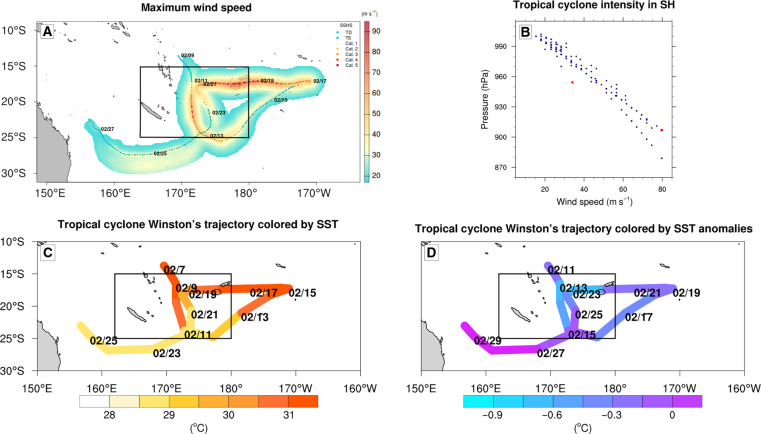
Tropical cyclone Winston. (**A**) Trajectory of the tropical cyclone Winston color-coded by its maximum sustained wind speed (in meters per second) and Saphir-Simpson Hurricane Scale (SSHS). Winston developed north of 15°S in early February 2016, and traveled through the study area from 11 to 27 February 2016. The contour envelope surrounding the trajectory indicates the wind speed calculated by a parametric cyclone model, delineating the cyclone’s zone of influence. This figure was produced using the R package StormR. (**B**) Scatter plot showing the minimum pressure (in hectopascals) versus the maximum sustained wind speed (in meters per second) for all Southern tropical cyclones recorded by the United States Agency. Data were extracted from the International Best Track Archive for Climate Stewardship (IBTrACS) database. The red dot and red star show respectively the intensity of tropical cyclone Winston in IBTrACS and ERA5 reanalysis. (**C**) Trajectory of the tropical cyclone (TC) Winston color-coded by sea surface temperature (SST; in degrees Celsius) 2 days before the TC and (**D**) the SST anomalies (in degrees Celsius) calculated as the difference between SST 2 days after the TC and 2 days before the TC.

This section delves into the climate phenomena responsible for the anomalies that triggered and dissipated the 2016 MHW. Figure 3A shows the MJO index from 1 January to 28 February 2016, illustrating a strong activity (MJO index greater than 1) across all phases of the MJO throughout the 2-month period. Figure 3B showcases the onset of the suppressed MJO phases (8, 1, and 2) in the western Indian Ocean by the end of December 2015, characterized by positive outgoing longwave radiation (OLR) anomalies and negative 850-hPa zonal winds anomalies. These signals propagate eastward, reaching the Southwest Pacific in early January. Consequently, the study area (black box in Fig. 1F) exhibited positive intraseasonal OLR anomalies and negative surface wind speed anomalies during January (Fig. 3, C and D), consistent with MJO phases 8, 1, and 2 (fig. S7), known for their convection-suppressing and wind speed–reducing effects in this area. This suppressed convection favors clear skies, leading to increased solar heat absorption, and lower winds favor reduced latent heat losses. Subsequently, positive-specific humidity anomalies following the MJO with a 15-day lag, transported by the anomalous circulation, emerge in the study area. These anomalies persist for 20 days during phases 3 and 4 of the MJO, further contributing to reduced latent heat loss as wind speeds return to near-normal levels (fig. S8). These results collectively indicate that this strong MJO event substantially contributed to the shortwave radiation and latent flux anomalies that led to surface warming during the 2016 MHW. Moving into February, MJO phases 4 to 7 are active, inducing enhanced convection in the study area characterized by negative OLR anomalies. Furthermore, the exceptionally warm waters combined with enhanced convection and low vertical wind shear (fig. S9) were favorable conditions for the formation of Cyclone Winston that transited through the study area from 9 to 27 February (*26*) (Fig. 4). Moreover, MJO phases 6 and 7 are known to increase the tropical cyclone frequency and intensity in the South Pacific (*27*, *28*), particularly during El Niño episodes (*29*), thus demonstrating the indirect effect of the MJO on the decline of the MHW. Winston first entered our study area on 9 February, which initially led to a positive wind stress anomaly triggering cooling by vertical mixing. Winston left our study area from 14 to 20 February; thus, we no longer captured the maximum wind speed but the mixing continued along its trajectory as the stratification has already been eroded. When Winston re-entered our study area on 20 February, it intensified strongly as it moved over very warm water near Fiji, and boasted among the strongest winds of all Southern tropical cyclones (Fig. 4, B and C), sharply intensifying surface wind speed and wind stress, resulting in increased oceanic heat loss via latent heat flux (Fig. 2, C and D). However, there is no jump in vertical mixing, as it moves over waters that were already mixed during its initial passage (fig. S10), making the mixing less efficient because the vertical temperature gradient has already been reduced.

### Why was the impact of the MJO so exceptional?

To elucidate the exceptional surface warming induced by the MJO during the austral summer of 2016, we investigated two hypotheses. First, during El Niño years, the study area typically experiences a small cooling of 0.2° to 0.5°C between July and December (figs. S1 and S2), resulting in reduced likelihood of MHWs (*15*, *19*). Thus, our first hypothesis examines whether the ocean preconditioning during El Niño 2016 differs from that of other El Niño years, potentially exhibiting no cooling or a warming. To assess this, we calculated the average temperature anomalies in the study area across all El Niño years. Our results reveal that from July to October 2015, temperatures are cooler than the average during typical El Niño years. This was followed by a warming trend from November to December 2015, albeit remaining within the range of other El Niño years. Therefore, we cannot conclude that surface ocean preconditioning was exceptional in 2015/2016.

**Fig. 5.**
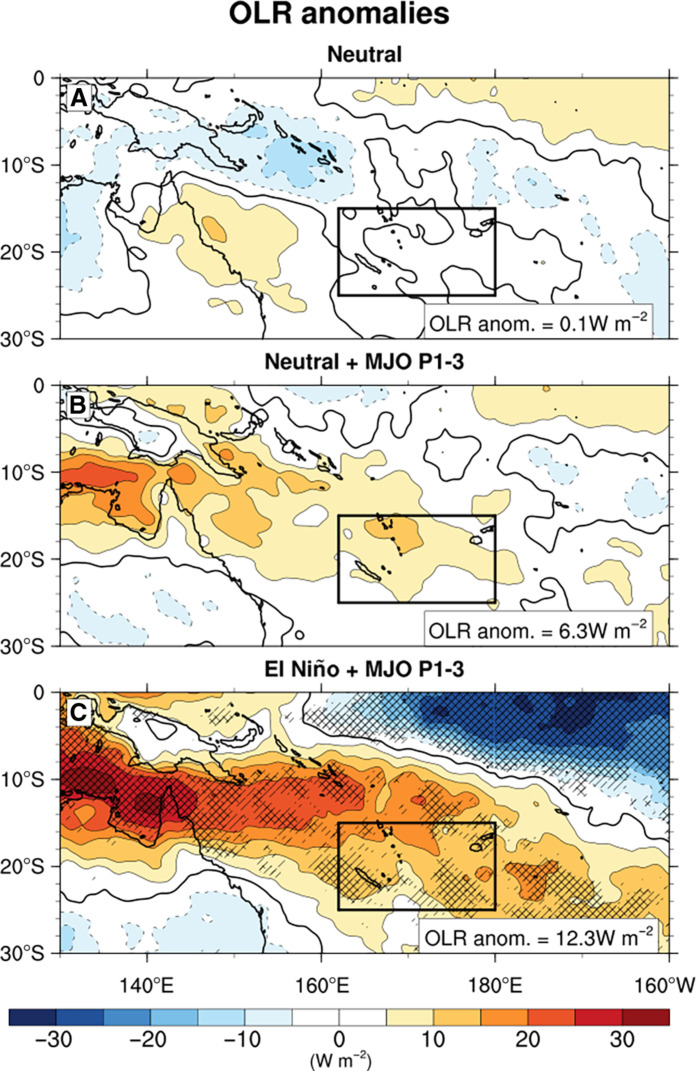
Madden-Julian Oscillation (MJO) and El Niño compound. Composites of austral summer (December-January-February) Outgoing Longwave Radiation (OLR) anomalies (shading in watts per square meter) without filtering interannual variabilities during (**A**) Neutral, (**B**) Neutral + MJO phases 1 to 3, and (**C**) El Niño + MJO phases 1 to 3. Black boxes show the region where marine heatwave (MHW) statistics and temperature budget were calculated. The spatial average of the OLR anomalies calculated in the study region is indicated on the bottom-right corner. The single and cross-hatched areas represent respectively the regions where the anomalies are significant at a threshold of 90 and 95% from a Kolmogorov-Smirnov nonparametric test. Data come from NOAA OLR daily data.

On the atmospheric side, El Niño is known to shift the SPCZ northwards (*18*, *22*), leading to reduced precipitation and increased solar radiation in the study area. While this typically warms surface waters, it is generally not sufficient to trigger a MHW, as evidenced by the occurrence of a MHW solely during the 2016 El Niño (Fig. 1). This prompts a second hypothesis related to a stronger role of atmospheric processes. The exceptional 2016 MHW event in the study area emerged as a compound event, combining atmospheric preconditioning by the 2016 El Niño event with a strong MJO, leading to increased positive OLR anomalies at the MJO-suppressed phase passage. Figure 5 shows the El Niño and Neutral (i.e. without El Niño or La Niña event) composites of OLR anomalies in austral summer and the combination with phases 1 to 3 of the MJO. Here, interannual variability has not been filtered out to demonstrate the combined effect of El Niño and the MJO. The difference between Neutral + MJO phases 1 to 3 and Neutral can be interpreted as the MJO effect. Likewise, the difference between El Niño + MJO phases 1 to 3 and Neutral + MJO phases 1 to 3 can be interpreted as the additional effect of El Niño. Figure 5 reveals that MJO phases 1, 2, and 3 increase OLR anomalies in the Southwest Pacific from 0.1 to 6.3 W m^−2^, while the effect of El Niño increases OLR by an additional 95%, rising from 6.3 to 12.3 W m^−2^ although this effect is only significant in the southern part of our study region (Fig. 5, B and C). This picture is also similar with the El Niño OLR anomalies (fig. S11). Furthermore, this spatial pattern of OLR anomalies was reinforced in 2016 by two concomitant factors. First, 2016 witnessed an extreme El Niño event, causing a more pronounced northward shift of SPCZ compared to moderate El Niño events (*18*, *22*). This resulted in a larger convection decrease in the Southwest Pacific and hence a stronger increase in solar radiation than during moderate El Niño years (fig. S11). Then, fig. S12 reveals that 2016 stood out among El Niño years for its exceptionally active MJO phases 1 and 2.

**Fig. 6.**
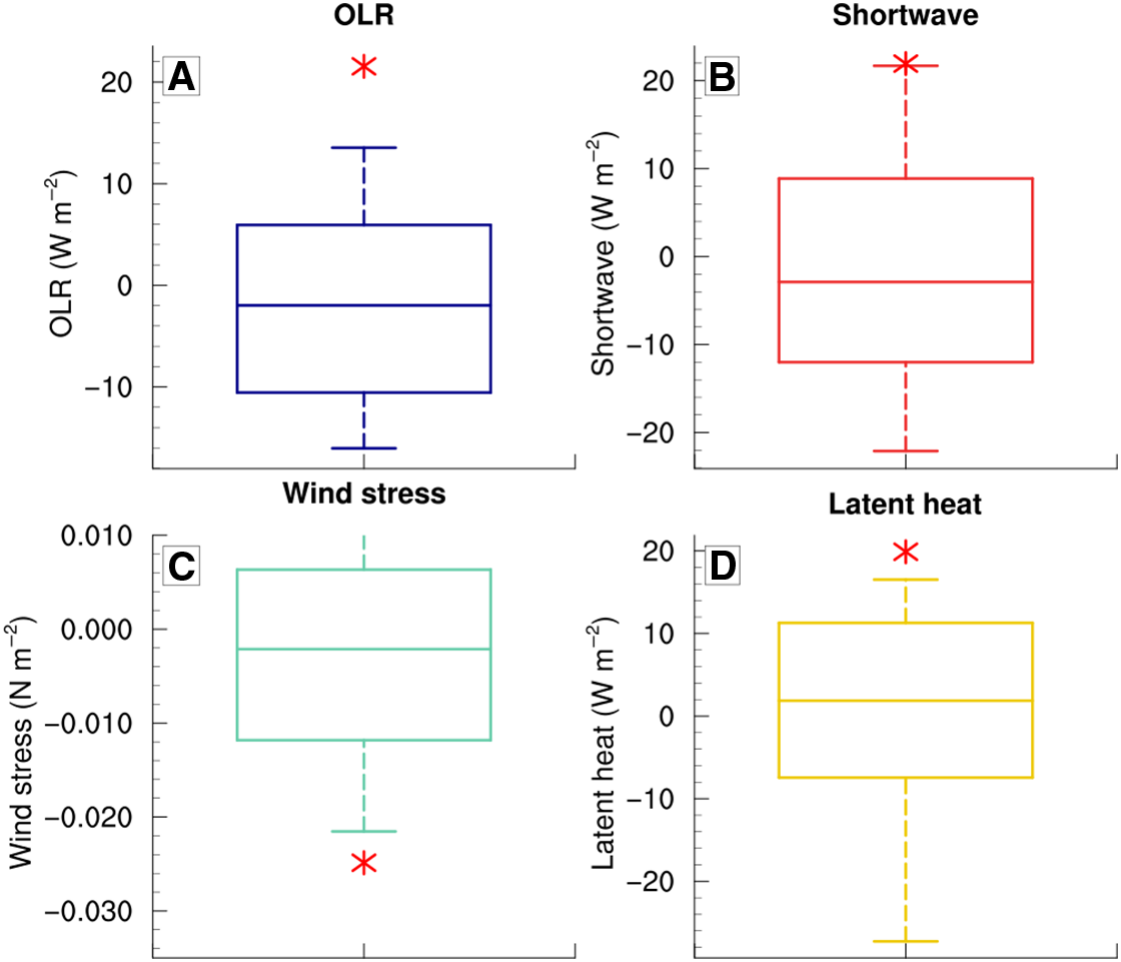
Distributions of the marine heatwave (MHW) drivers. Boxplots show the anomalies of (**A**) Outgoing Longwave Radiation (OLR) (in watts per square meter), (**B**) Shortwave radiation (in watts per square meter), (**C**) wind stress (newtons per square meter), and (**D**) Latent heat fluxes (in watts per square meter) for 1 January to 10 February that corresponds to the growth period of the 2016 MHW. The anomalies are calculated over the period 1993–2019. The boxplot bounds are the first (Q25) and the third (Q75) quartiles, the inside horizontal line shows the median (Q50), and the vertical segments are the first (Q10) and the last deciles (Q90). The red star shows the anomalies for 1 January to 10 February 2016.

Last, we have placed the main drivers of 2016 MHW into a broader context by comparing the 1 January to 10 February anomalies for OLR, shortwave radiation, wind stress, and latent heat fluxes with those over the 1993–2019 period (Fig. 6). This analysis reveals that 2016 anomalies are exceptional, falling above the 90th percentile or below the 10th percentile, for all these drivers.

## DISCUSSION

**Fig. 7.**
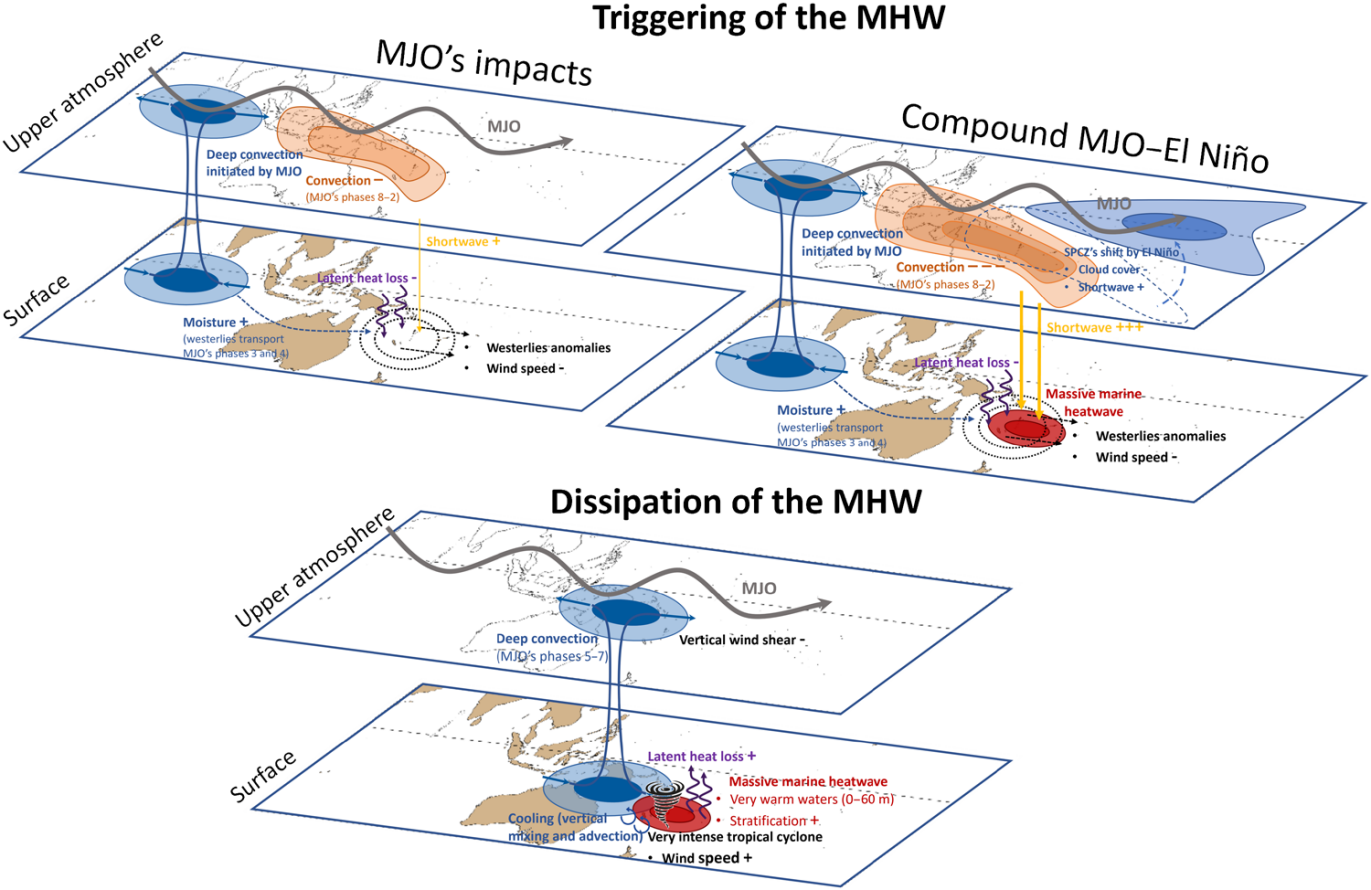
Schematic overview of the 2016 marine heatwave (MHW) life cycle. The upper panel shows the processes that triggered the 2016 MHW, including the effect of the Madden-Julian Oscillation (MJO) and the compound event “MJO-El Niño”. The bottom panel highlights the factors favorable to the genesis of tropical cyclone Winston and then its contribution to the rapid dissipation of the MHW. The + and − signs indicate respectively an enhanced and reduced effect.

The schematic in Fig. 7 provides a comprehensive overview of the mechanisms underlying the 2016 MHW. Originating from the Indian Ocean, a MJO event propagates eastward, eventually reaching the Southwest Pacific. During MJO phases 8, 1, and 2, suppressed convection and winds in this region lead to increased shortwave radiation and reduced evaporative cooling, resulting in a warmer SST. Subsequent MJO phases 3 and 4 witness moisture transport from anomalous circulation, further reducing evaporative cooling and thus warming the SST. Concurrently, the extreme El Niño event shifts the SPCZ northward by ~10°, exacerbating convection suppression and SST warming. As the atmosphere moisture increases, convection resumes during MJO phases 4 to 7. This favorable environment, characterized by extremely warm waters, atmospheric convection, and low vertical wind shear, facilitates the genesis of the most intense tropical cyclone recorded in the southern hemisphere. Its extreme winds prompted rapid surface water cooling through evaporative cooling and vertical ocean processes (advection and turbulent mixing). The life cycle of the 2016 MHW in the Southwest Pacific thus emerges as the outcome of rare and complex interactions between two large-scale climate modes, the MJO and the extreme El Niño event, alongside the influence of a very intense tropical cyclone. This study illustrates, through a real-world case, how all these diverse climatic phenomena can interact to both trigger and dissipate a MHW.

Nevertheless, some studies have already discussed the individual influence of the MJO, El Niño–Southern Oscillation (ENSO), and tropical cyclones on MHWs in the Pacific. For instance, a recent study has demonstrated that the increased latent heat fluxes during the MHWs favor more intense tropical cyclones (*21*). At the intraseasonal timescale, the phases 2 to 5 of the MJO suppress the convective activity while promoting the Ekman pumping off the northwest coast of Australia, leading to warmer SSTs and a higher occurrence of MHWs in that region (*30*, *31*). ENSO also emerges as a major driver of MHW likelihood within the Pacific. During El Niño events, elevated SSTs in the central and eastern tropical Pacific promote MHWs there (*15*, *32*). Conversely, La Niña events elevate SSTs in the Southwest Pacific, thereby increasing the likelihood of MHWs in the region (*15*, *19*). Consequently, the phase of ENSO emerges as a critical factor in either promoting or suppressing MHW occurrences across various regions. However, as illustrated here, the interactions between large-scale climate modes can reshape the conventional MHW understanding associated to ENSO, potentially avoiding misinterpretation and forecasting errors.

### Seasonal and short-term forecasting

Seasonal forecasting of MHWs is gaining traction among managers and stakeholders (*4*, *33*). Dynamical forecasts (*34*–*36*), based on numerical models, predict environmental conditions from days to seasons ahead, relying on accurate observations for initialization and physical process representations (*37*). Improving model parameterizations for both the atmosphere and ocean is crucial for simulating MHW-driving air-sea fluxes, and multimodel ensembles reduce parameterization uncertainty (*34*). Statistical forecast systems, using machine learning, exhibit good skill at short lead times (*38*, *39*) (up to 7 days) but may face challenges in warmer oceans due to potential nonstationarity and because these models are trained on past conditions (*37*). MHW complexity and nonstationarity pose challenges, especially when rare event combinations occur as the 2016 MHW in the Southwest Pacific (*40*). Other methods derive forecast skill from a better understanding of the drivers of MHWs. For instance, Wang *et al.* (*41*) showed that a combination of positive Indian Ocean Dipole and La Niña increases the MHWs likelihood in the following months in Western Australia. Their results suggest that the future occurrence of MHWs can be assessed by monitoring the climate modes driving them. This alternative approach to MHW seasonal forecasting becomes valuable when driver forecast skill is high or direct MHW forecast products are unavailable. Here, we show that MHWs can be triggered and stopped by the interplay of multiple climate phenomena, which present varying levels of predictability at different timescales: Seasonal forecast systems can be skillful at lead times of a few months for ENSO and a few weeks for the MJO (*42*), while tropical cyclones are notoriously difficult to predict more than a few days in advance (*43*). Therefore, in our case, we might expect that the event development may have been forecasted some weeks in advance but not its decay. This would need further investigation in available retrospective seasonal forecasts.

### Climate change

Climate change likely affected the characteristics of this event as our target region has experienced a long-term warming trend of ~0.2°C decade^−1^ over the past three decades (fig. S13), with the 2016 MHW occurring at the end of the 1993–2022 period. To analyze the role of climate change, we applied the detection method to linearly detrended SST data (fig. S14). When the warming trend is removed, the 2012 and 2022 MHWs occurring during La Niña years are no longer detected. In addition, it modifies the characteristics of the 2016 MHW as follows: Its spatial extent is reduced to 65% of the study region, the intensity remains similar, and the duration is strongly reduced to 8 days. This analysis reveals the influence of global warming on the features of 2016 MHW, whose exceptional spatial extent and duration is partly driven by the climate change warming trend.

Frölicher et al. (*6*) showed in global climate models that MHW frequency, intensity, and duration are projected to uniformly increase by a factor of 10 to 40 by the end of the 21st century, depending on the emission scenario considered, with the strongest changes occurring in the tropical regions. These changes are mainly driven by the long-term temperature trend, although natural variability such as ENSO and MJO can either damp or amplify the MHW likelihood. Studies about changes in MJO features consistently project an increase of MJO amplitude and phase speed (*44*–*48*). This will result in stronger but less persistent OLR anomalies, leading to a contrasting influence on MHWs. In addition, numerous studies have also analyzed possible ENSO changes in the future and, despite ongoing debate about the reliability of ENSO projections due to Coupled Model Intercomparison Project (CMIP) model biases (*49*–*51*), many of them indicate a frequency increase of extreme El Niño events (*52*, *53*), extreme La Niña events (*54*, *55*), and multiyear La Niña events (*56*). Given the association of extreme El Niño events with extreme northward shifts of the SPCZ and La Niña events with warmer waters in the Southwest Pacific, their frequency is anticipated to amplify the MHW frequency and intensity in this region. Last, projections indicate a decrease in the frequency of tropical cyclones in the Southwest Pacific in response to the strengthening of vertical wind shear (*57*–*59*). Consequently, the MHW dissipation effect of tropical cyclones highlighted here could become less frequent in the future. Nevertheless, projections of intense tropical cyclones remain highly uncertain because of the climate models limited accuracy to model them, potentially influencing this trend.

### Ecological impacts

The February 2016 MHW was the first reported event to trigger a massive bleaching episode on New Caledonia’s coral reefs (*8*), leading to 87% bleaching with 90% affected between 1- and 5-m depth. Coral bleaching, primarily triggered by prolonged warm waters, is exacerbated by high ultraviolet (UV) radiation levels (*60*). The 2016 MHW, characterized by UV radiation levels 10% higher than normal for at least a month and a half (*8*), likely increased the risk of bleaching. This was due to the combination of a passing MJO event atop the 2016 extreme El Niño, suppressing convection and thus increasing solar flux, leading to that exceptional bleaching event. Consequently, this MHW had a greater ecological impact compared to previous events where these two climate modes did not combine, underscoring the significance of extreme events where multiple processes contribute to extreme outcomes. Concurrently, during the same period, a 22% decrease in chlorophyll-a concentration occurred in the region (fig. S15). This compound event of high temperature and low chlorophyll may have had cascading effects up the trophic chain, potentially contributing to the massive fish kills observed in Vanuatu, Fiji, and Kiribati (*11*). In addition, it might have prompted the migration of some pelagic fish species in response to their thermal tolerance or food availability (*61*, *62*). While identifying the full causes of these impacts falls beyond the scope of this article, further study would be necessary to comprehensively understand their causes.

In summary, our findings underscore the exceptional nature of this MHW life cycle, notable as the sole occurrence during an El Niño event in the Southwest Pacific and involving the interaction between two large-scale climate modes. This interaction resulted in reduced cloud coverage, wind suppression, and air moistening leading to anomalous positive heat gain in the ocean mixed layer. The combination of this extreme temperature event with strong UV radiation led to a massive bleaching event, while the compound with low chlorophyll a likely may have increased the mortality of many fish species among the Melanesian islands. Furthermore, our study demonstrates that the MHWs in the Southwest Pacific are the result of many potentially nonlinear interacting processes, with no straightforward single mechanism that would allow to easily forecast such events in the future or deduce their probability in future climates from model projections. Thus, our study demonstrates the need for more research on the dominant large-scale climate modes and their temporal and spatial interferences.

## MATERIALS AND METHODS

### Data

The MHWs were analyzed using the daily National Oceanic and Atmospheric Administration (NOAA) Optimum Interpolation (OI) Sea Surface Temperature (SST) V2 High Resolution Dataset at 0.25° resolution (*63*) for the period 1993–2022. To compute MJO index and composites of the atmospheric variables we used the fifth generation of European Center for Medium-Range Weather Forecasts (ECMWF) reanalysis (ERA5) (*64*). Daily values were obtained by averaging the 6-hour data of the OLR and the zonal and meridional components of the wind at 10 m, at 850 hPa, and at 200 hPa. The interpolated NOAA OLR daily data (*65*) were also used as proxy of the convection. The trajectory, maximum sustained wind speed, and minimum pressure for Winston tropical cyclone were extracted from the International Best Track Archive for Climate Stewardship (*66*) database.

For analyzing the mechanisms at the origin of MHW we used two global ocean simulations. First, GLORYS performed by the Copernicus Marine Environmental Monitoring Service and on the basis of Nucleus for European Modeling of the Ocean (NEMO) model (*67*), with a horizontal resolution of 1/12° and 50 vertical levels. GLORYS is forced at the surface by the ECMWF ERA-Interim atmospheric reanalysis (*68*). GLORYS outputs are available from 1993 to 2019, during which the model assimilates along-track satellite altimetry, satellite SST, sea ice concentrations, and in situ profiles of temperature and salinity from the Coriolis Ocean database ReAnalysis dataset. Because of large known biases in radiative fluxes at the surface in ERA-Interim, a satellite-based large-scale correction is applied toward the NASA/GEWEX Surface Radiation Budget 3.0/3.1 product (*69*). Thus, for analyzing the surface heat fluxes we used the forcing fields of GLORYS. The second global ocean simulation is also based on NEMO model at 1° horizontal resolution with a meridional resolution refinement to 1/3° in the equatorial band and 75 vertical levels and spanning the period 1958 to 2022 (*70*). This oceanic simulation is forced at the surface by the second Japanese global reanalysis dataset called JRA-55 (*71*, *72*). No assimilation is carried out; however, an online mixed-layer temperature budget is implemented. Chlorophyll-a concentrations come from the Copernicus Marine service, and we used the daily “cloud free” product at 4-km spatial resolution (*73*).

### MHW statistics

Following Hobday *et al.* (*1*), at each grid point, a MHW event is detected when the SST exceed the seasonally varying 90th percentile for at least 5 days with as reference the period 1993–2022. Then, a MHW is detected during the austral summer season [December-January-February (DJF)] in the Southwest Pacific domain (162°E to 180°E; 25°S to 15°S) when 50% of its grid points are covered by a MHW during at least 5 days. Detection is interrupted when the surface falls below this threshold. Last, we calculated the duration, intensity, and the maximum surface cover of each MHW event. The intensity of a MHW is calculated by averaging the temperature anomalies of all grid points in the study area where a MHW has been detected. The duration corresponds to the number of days when 50% of the study region is covered by a MHW.

### Vertical extent

MHWs were identified independently at each depth level and for each grid cell available in GLORYS from surface to 1500 m. The maximum depth of detection, from the surface to depth without interruption, was recorded as the vertical extent of the surface MHW event. Then the vertical extent has spatially averaged over our domain.

### Upper ocean temperature budget

A vertical averaged temperature budget of upper ocean is used to determine the relative roles of air-sea heat fluxes (first term at the right of Eq. 1), horizontal advection (second term at right of Eq. 2) and residual term (third term at right of Eq. 1) to the warming of seawater temperature during the austral summer of 2016 in the Southwest Pacific. The temperature budget is defined as

$$\underset{\text{Tendency}}{\underbrace{\frac{\partial T}{\partial t}}}=\underset{\text{Net heat flux}}{\underbrace{\frac{Q_{\mathrm{net}}-Q_{h}}{\rho_{0}C_{p}h}}}-\underset{\text{Horizontal advection}}{\underbrace{\left\langle u\frac{\partial T}{\partial x}+v\frac{\partial T}{\partial y}\right\rangle }}-\underset{\text{Residual}}{\underbrace{\varepsilon }}$$ (1)

where angle brackets denote the volume average over the 20-m-layer depth *h* and the Southwest Pacific area (162°E to 180°E; 25°S to 15°S) (black box in Fig. 1F), ρ_0_ is the seawater density, *C*_*p*_ is the seawater specific heat, *T* is the seawater temperature, *u* and *v* are respectively zonal and meridional currents, *Q*_*net*_ is net surface heat fluxes, *Q_h_* is the part of surface solar heat flux that radiates out at the base of layer *h* (here 20 m), and ε is the residual term (see Eqs. 2 and 3 below). The temperature budget is calculated at each depth and then is averaged over a depth *h* of 20 m chosen close to the mixed-layer depth (fig. S4)

### Decomposition of the surface heat fluxes

The net surface heat flux reaching the sea surface (*Q*_*net*_) can be decomposed as turbulent fluxes which are the sum of latent heat flux (*Q*_*lh*_), sensible heat flux (*Q*_*sh*_), and radiative forcings: the sum of the net surface shortwave radiation (*Q*_*sw*_, which is the incoming solar minus outgoing reflected shortwave due to albedo) and the net surface longwave radiation (*Q*_*lw*_).

$$Q_{\text{net}}=Q_{\text{lh}}+Q_{\text{sh}}+Q_{\text{sw}}+Q_{\text{lw}}$$ (2)

Some of *Q*_*sw*_ radiates out of the base of the 20-m layer. The depth propagating radiative forcing (**Q*_*h*_*) is calculated according to Paulson and Simpson (*74*) with the two extinction lengths for medium turbidity Jerlov water type IB

$$Q_{h}=Q_{\text{sw}}\left[Re^{-h/\xi_{0}}+\left(1-R\right)e^{-h/\xi_{1}}\right]$$ (3)

where *h* is the layer from surface to 20 m, *R* = 0.67, ξ_0_ = 1.0 m, and ξ_1_ = 17 m. Therefore, the shortwave radiation stored in the layer *h* is *Q*_*sw*_*-*Q*_*h*_*.

### MJO diagnostics

MJO diagnostics calculated here follows US Climate Variability and Predictability MJO working group recommendations (*75*) MJO index was calculated from daily OLR and zonal wind velocity at 850 hPa and at 200 hPa extracted from ERA5 reanalysis. First, daily anomalies were calculated by subtracting the daily climatologies. Then, intraseasonal anomalies were constructed by applying a Lanczos band-pass filter to keep only frequencies between 20 and 100 days. Last, the wind and OLR-filtered fields were normalized by the square root of the zonal mean of their temporal variance and were equatorial-averaged (15°S to 15°N) before input into the covariance matrix used to conduct the Empirical Orthogonal Function (EOF) analysis. The two leading multivariate EOFs were used to derive a composite MJO life cycle for January to February as described by Wheeler and Hendon (*76*). The MJO was defined to be strong during periods when PC1^2^ + PC2^2^ exceeds 1, and these periods of high amplitude were retained in the composite analysis. For each phase, composites were generated by averaging across all days that exceed the specified amplitude threshold. In Fig. 5, the composite fields were calculated from unfiltered data to keep the interannual variability.

### El Niño–Southern Oscillation

El Niño (La Niña) events are defined as austral summer seasons (DJF) when the DJF Niño3.4 SST anomaly is greater (lower) than 0.5°C, following NOAA’s index: www.cpc.ncep.noaa.gov/products/analysis_monitoring/ensostuff/ONI_v5.php.

### SST trend calculation

The monthly average of SST is computed from the daily values. Then, at each grid point, the linear trend is computed with the Theil-Sen estimator (*77*, *78*). The trend computed with this method is the median of the slopes determined by all pairs of sample points. The advantage of this method is that it is much less sensitive to outliers; thus, the extreme values will have less influence on the trend calculation than with the least squares method. The trends are computed seasonally. The significance of trends is evaluated from a Mann-Kendall nonparametric test with a threshold of 95%.
