# Supplementary material for: The massive 2016 marine heatwave in the Southwest Pacific: An “El Niño–Madden-Julian Oscillation” compound event
Source: Sci Adv. 2024 Oct 9;10(41):eadp2948. doi: 10.1126/sciadv.adp2948 (PMC11463258; doi:10.1126/sciadv.adp2948)
Supplement: Supplementary file 1 — Figs. S1 to S15 [file sciadv.adp2948_sm.pdf]

Science Advances
AAAS

Supplementary Materials for

The massive 2016 marine heatwave in the Southwest Pacific: An "El Niño–Madden-Julian Oscillation" compound event

Cyril Dutheil *et al.*

Corresponding author: Cyril Dutheil, cyril.dutheil@ird.fr

Sci. Adv. **10**, eadp2948 (2024)
DOI: 10.1126/sciadv.adp2948

This PDF file includes:

Figs. S1 to S15

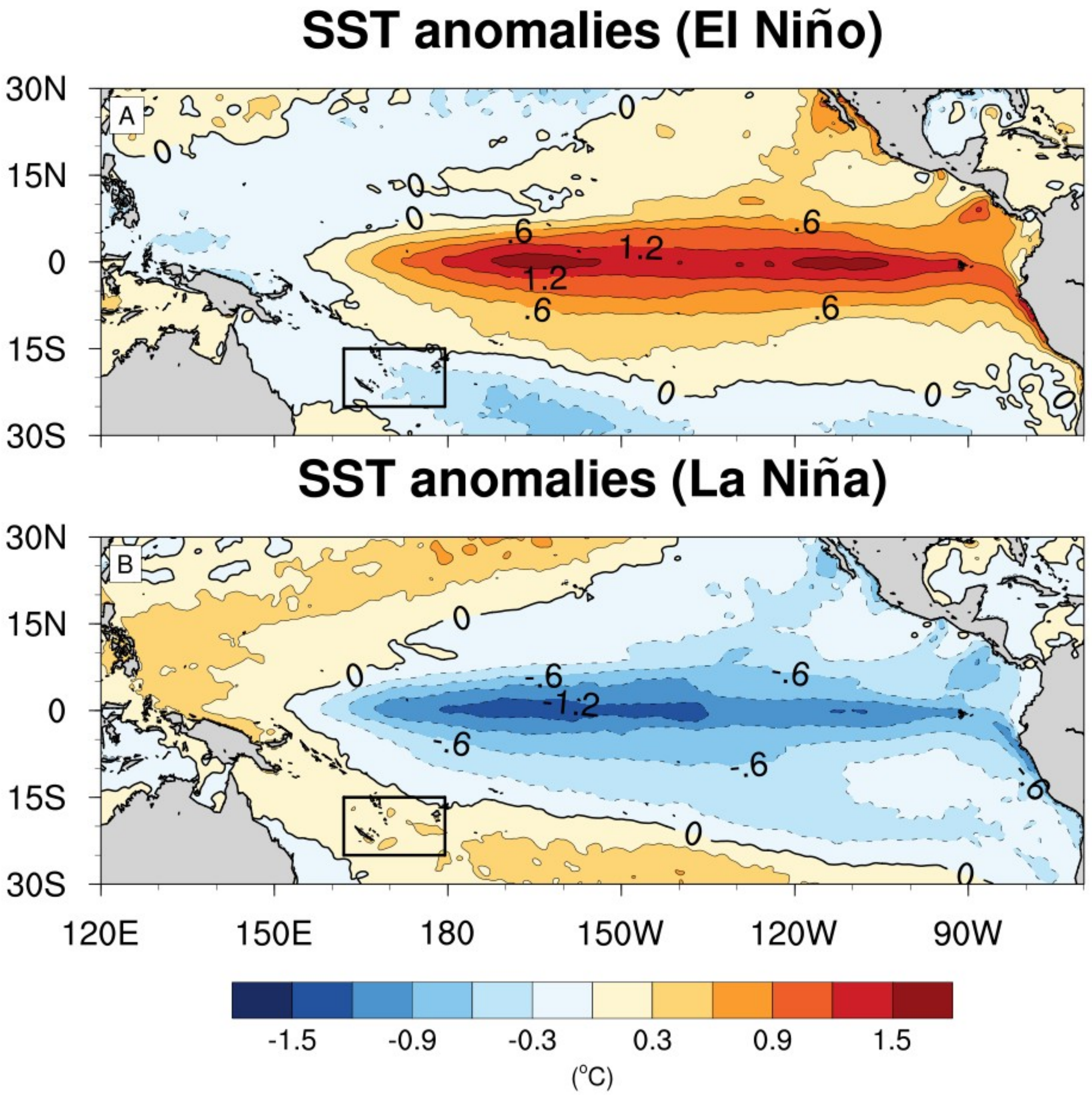

Fig. S1 : Composites of austral summer sea surface temperature anomalies during **(A)** El Niño and **(B)** La Niña years. The black box shows the study area [162°E-180°E; 15°S-25°S] used for the marine heatwave statistics and the temperature budget calculation.

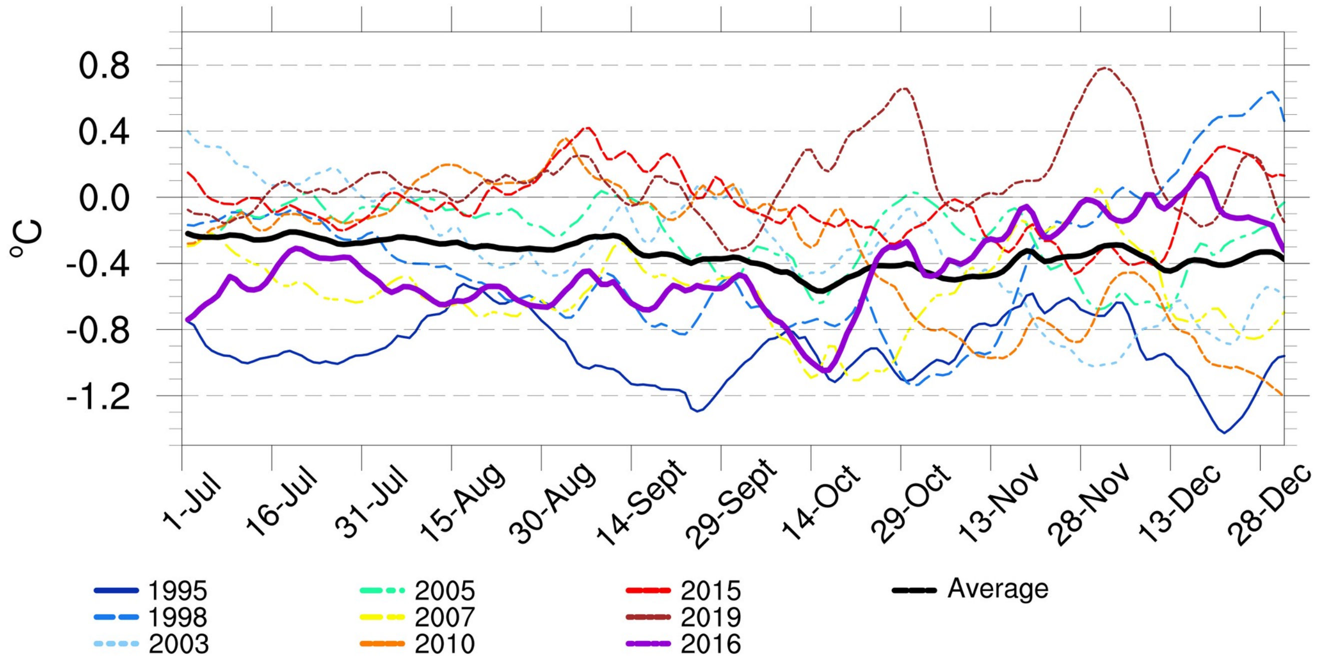

Fig. S2. Time series of sea surface temperature anomalies between July and December in the 0-20m layer averaged over our region (black box in Fig. 1F) for all El Niño years between 1993 and 2019. The colored lines show each year individually, and the black line is the average of all El Niño years. The years are labeled by their El Niño years, which means that the 2016 label corresponds to the period 1st July to 31st December 2015 for instance.

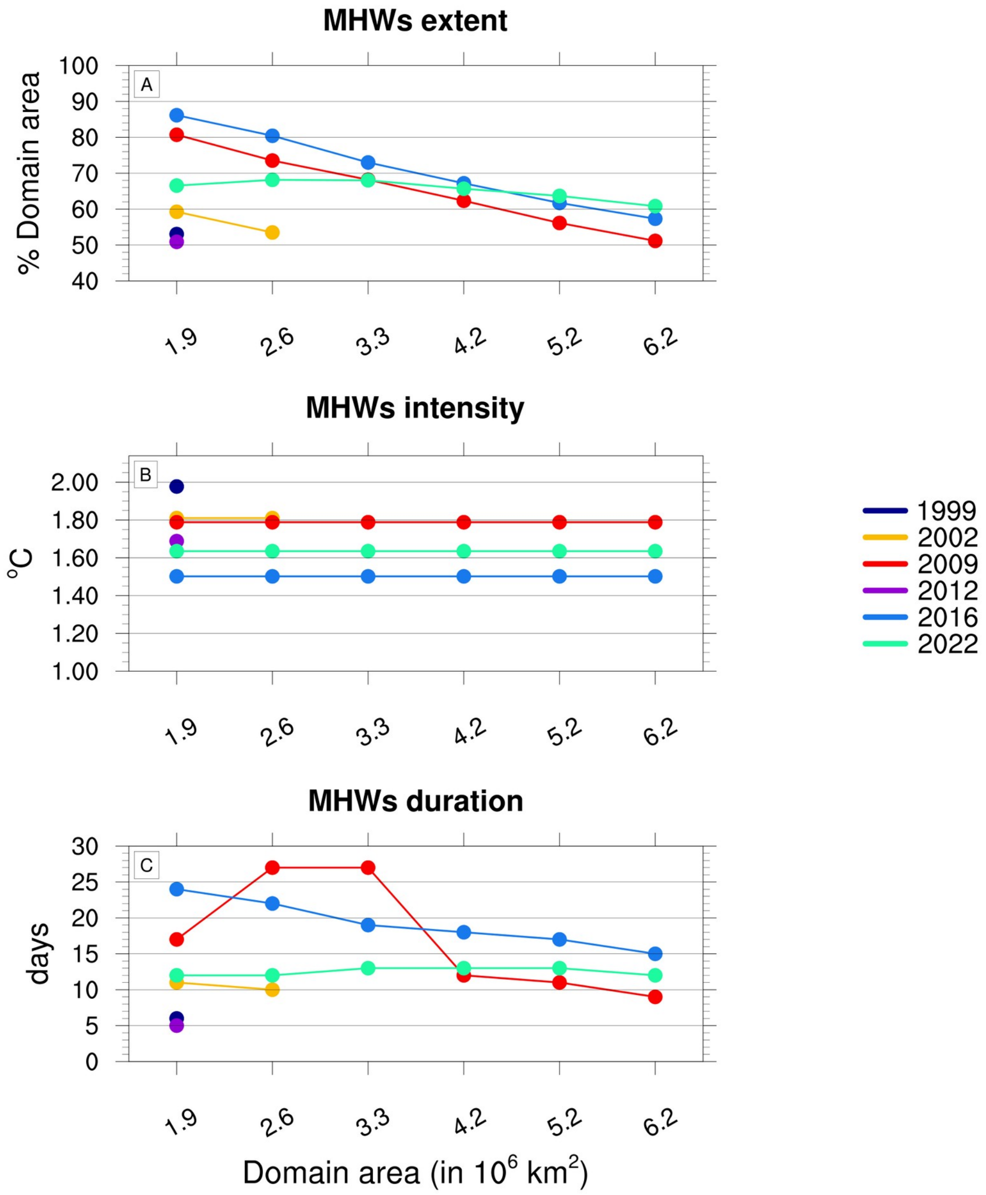

Fig. S3 : Statistics of the six largest marine heatwaves (MHWs) identified in Fig. 1 according to domain area. **(A)** The maximum proportion of our study area experiencing MHW conditions (in % of grid cells), **(B)** maximum intensity (in °C) and **(C)** the duration of MHWs (in days). A MHW is detected when the event covers 50 % of our study region during at least 5 days. The lowest area corresponds to that shown in Fig. 1. The following areas are obtained by enlarging each limit by 1°.

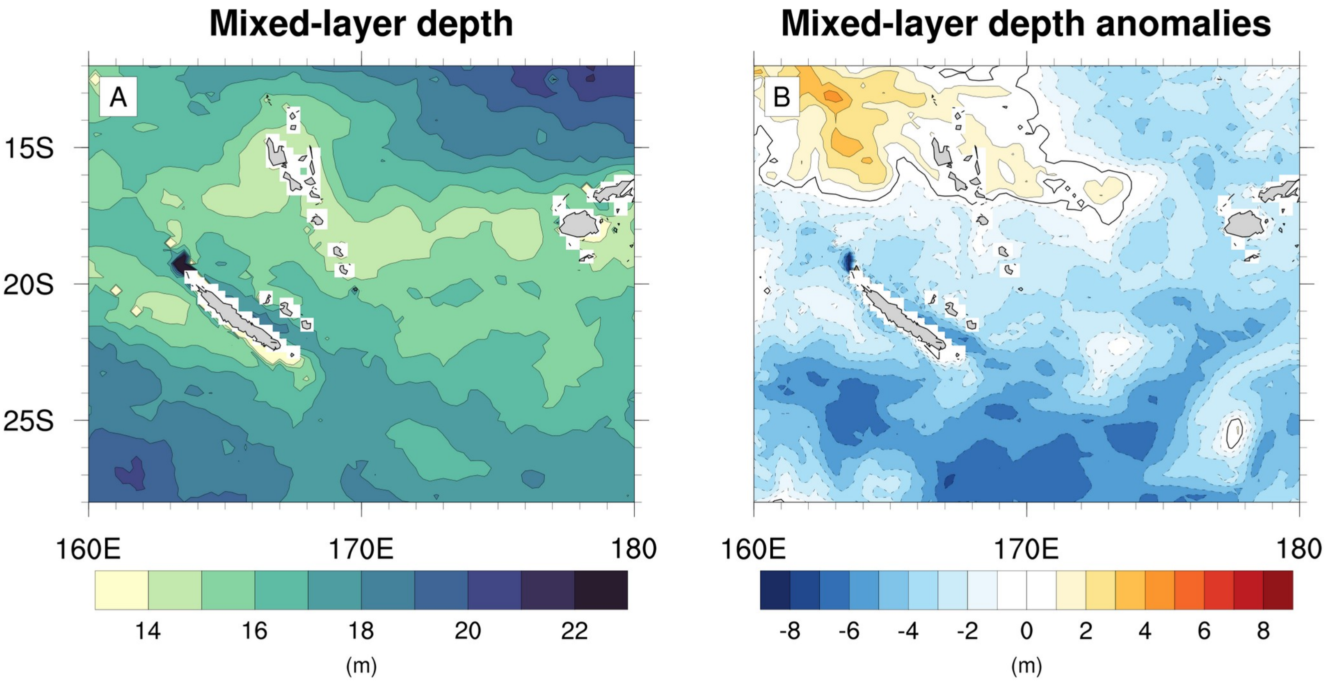

Fig. S4 : (A) Climatology and **(B)** anomalies of the mixed-layer depth (in m) from 01st January to 15th February 2016

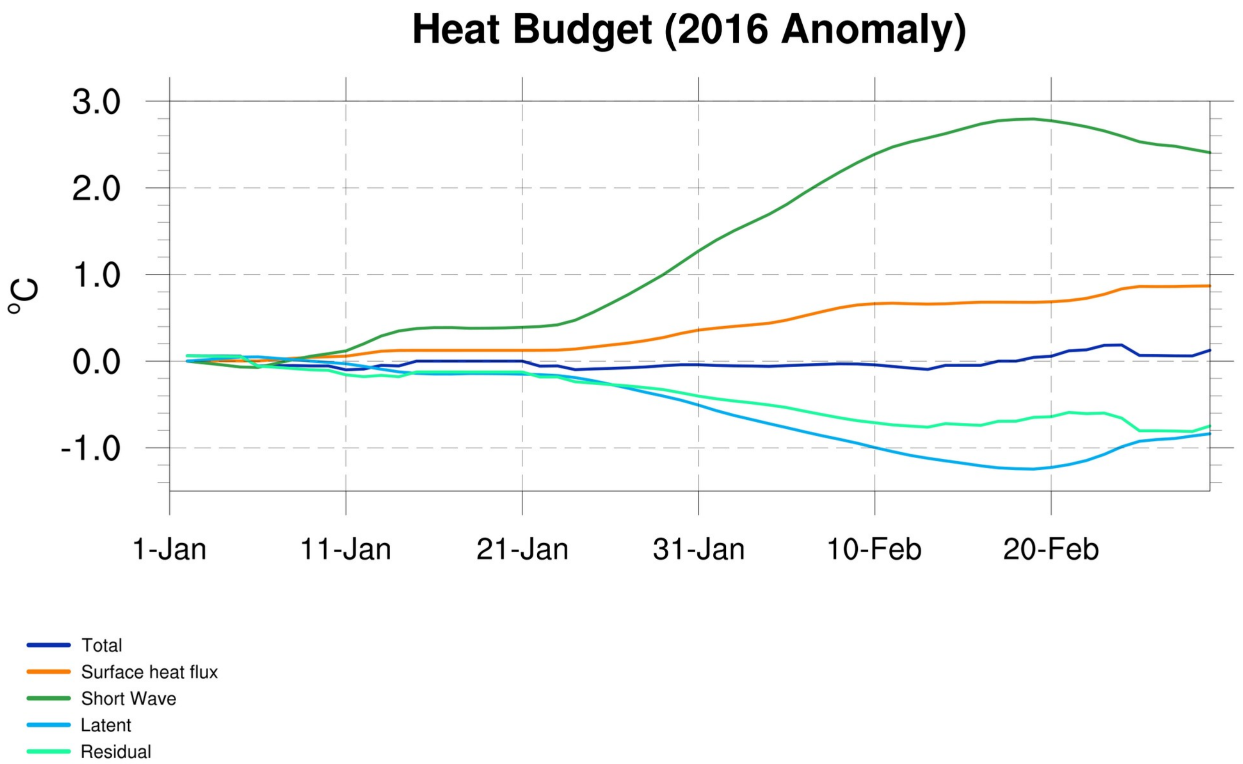

Fig. S5 : Difference between the time-integrated mixed layer temperature budget calculated on the actual mixed layer depth and the climatological mixed layer depth from January 2016 to February 2016 (in °C). Blue line represents temperature, the orange line is the contribution of air-sea heat flux, dark green line is the contribution of the shortwave radiation, light blue line is the contribution of the latent heat flux and the light green line is the residual. Heat budget is calculated from GLORYS.

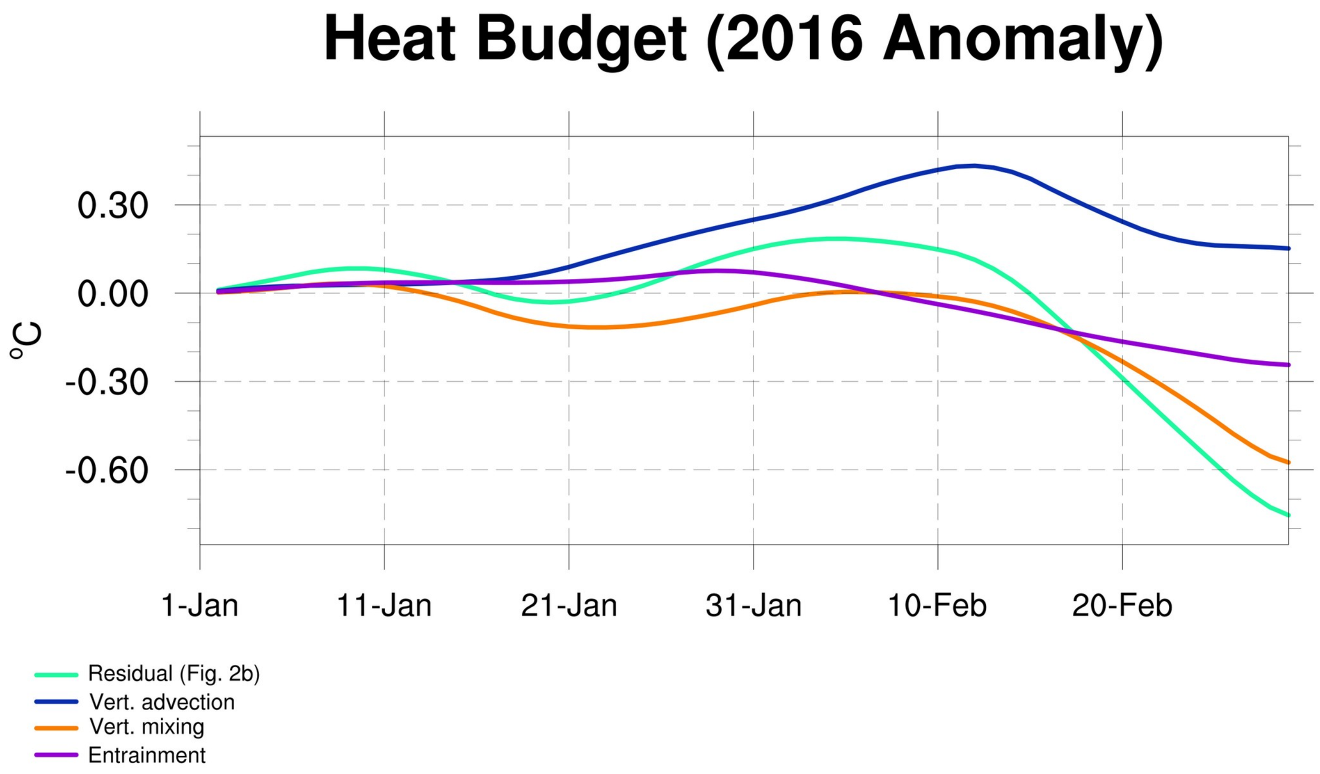

Fig. S6: Decomposition of the residual term showed in dashed green line Fig. 2B. The blue line represents the contribution of the vertical advection, the orange line is the contribution of the vertical mixing and the purple line is the contribution of the entrainment at the base of the mixed layer. Data come from a NEMO simulation with online mixed layer temperature budget calculation.

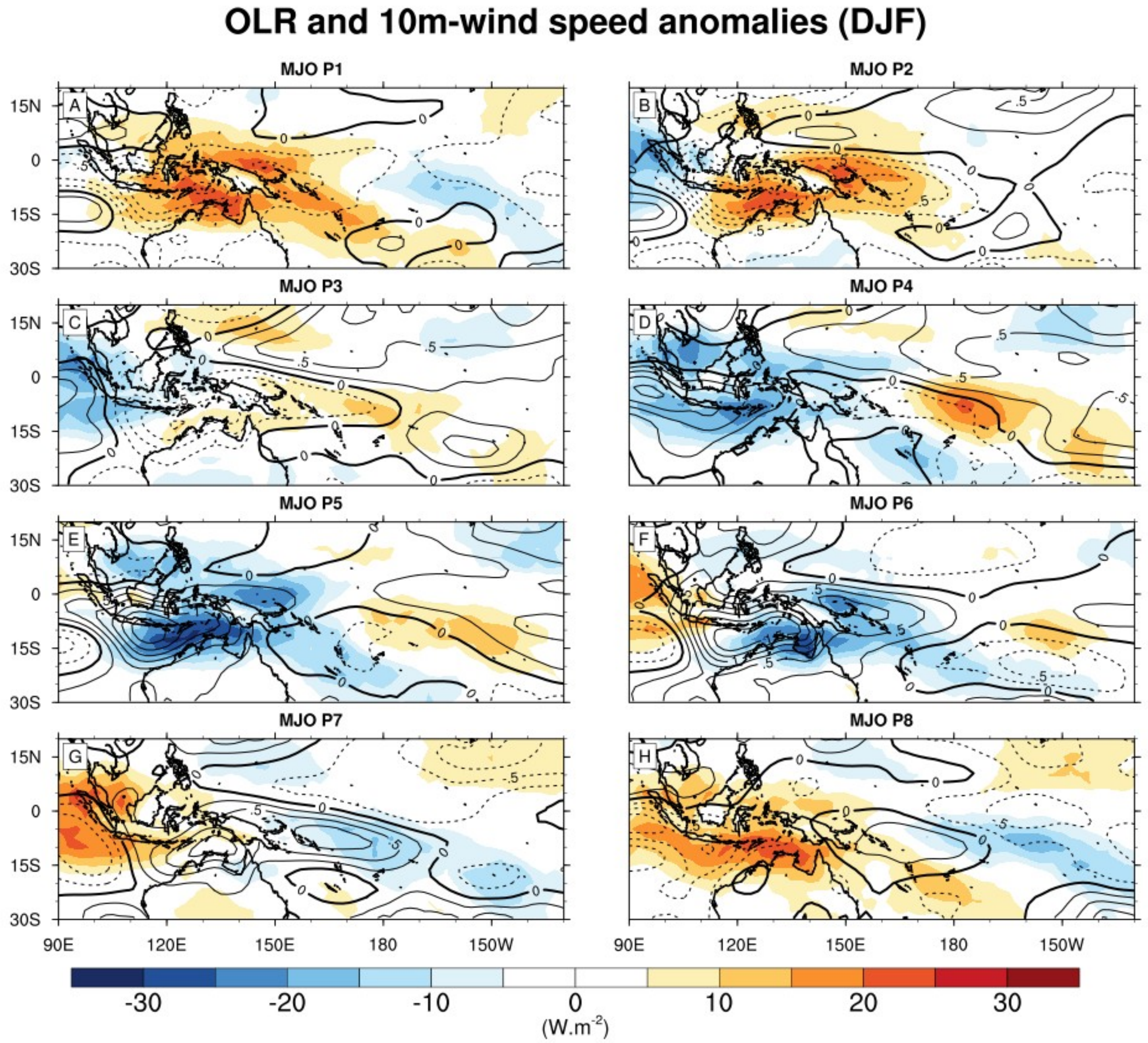

Fig. S7 : Austral summer (December-January-Febraury) Outgoing Longwave Radiation (OLR, shading ; in $W.m^{-2}$) and 10m-wind speed (contour ; in $m.s^{-1}$; contours lines with $0.25 m.s^{-1}$ interval and negative values dashed) anomalies from ERA5 reanalysis during the Madden Julian Oscillation phases **(A)** 1, **(B)** 2, **(C)** 3, **(D)** 4, **(E)** 5, **(F)** 6, **(G)** 7, and **(H)** 8.

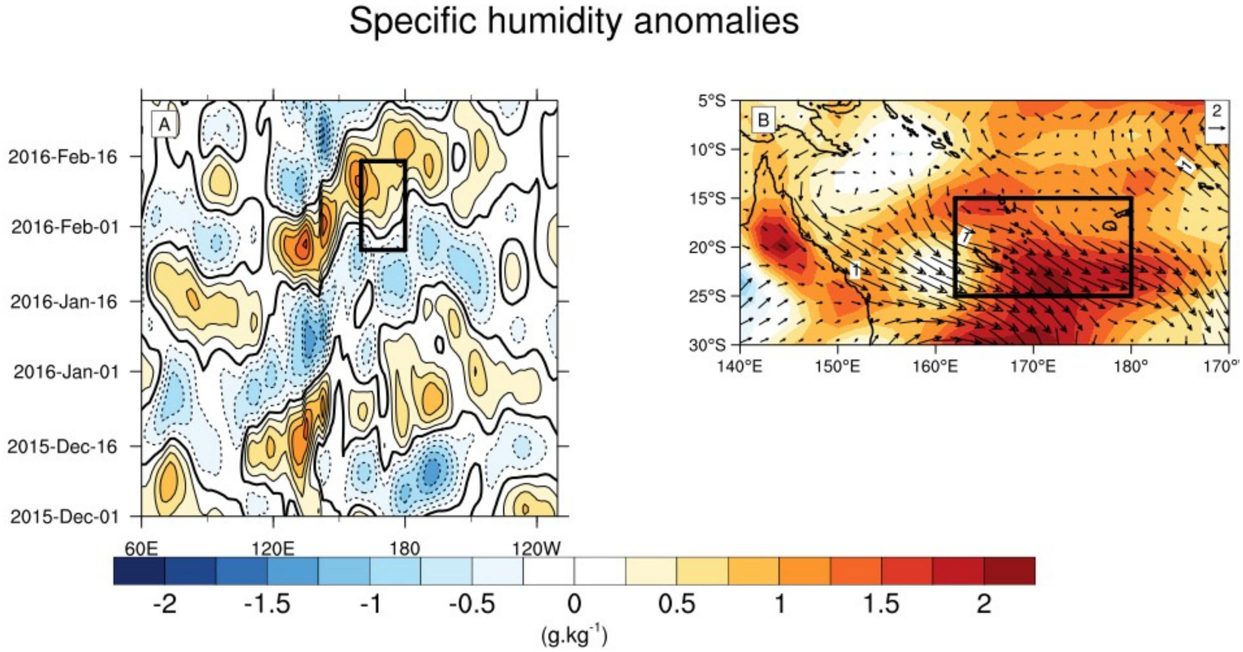

Fig. S8 : (A) Hovmöller diagram of intraseasonal specific humidity (in g.kg^{-1}) anomalies averaged between 20°S and 0°N for the austral summer (December-January-Febraury) 2016. The black box indicates the period from 22nd January to 15th February within the region [160°E-180°]. **(B)** Map of the specific humidity (shading ; in g.kg^{-1}) and surface wind (vectors, in m.s^{-1}) anomalies from 22nd January to 15th February. The black box shows the region [160°E-180°,25°-15°S].

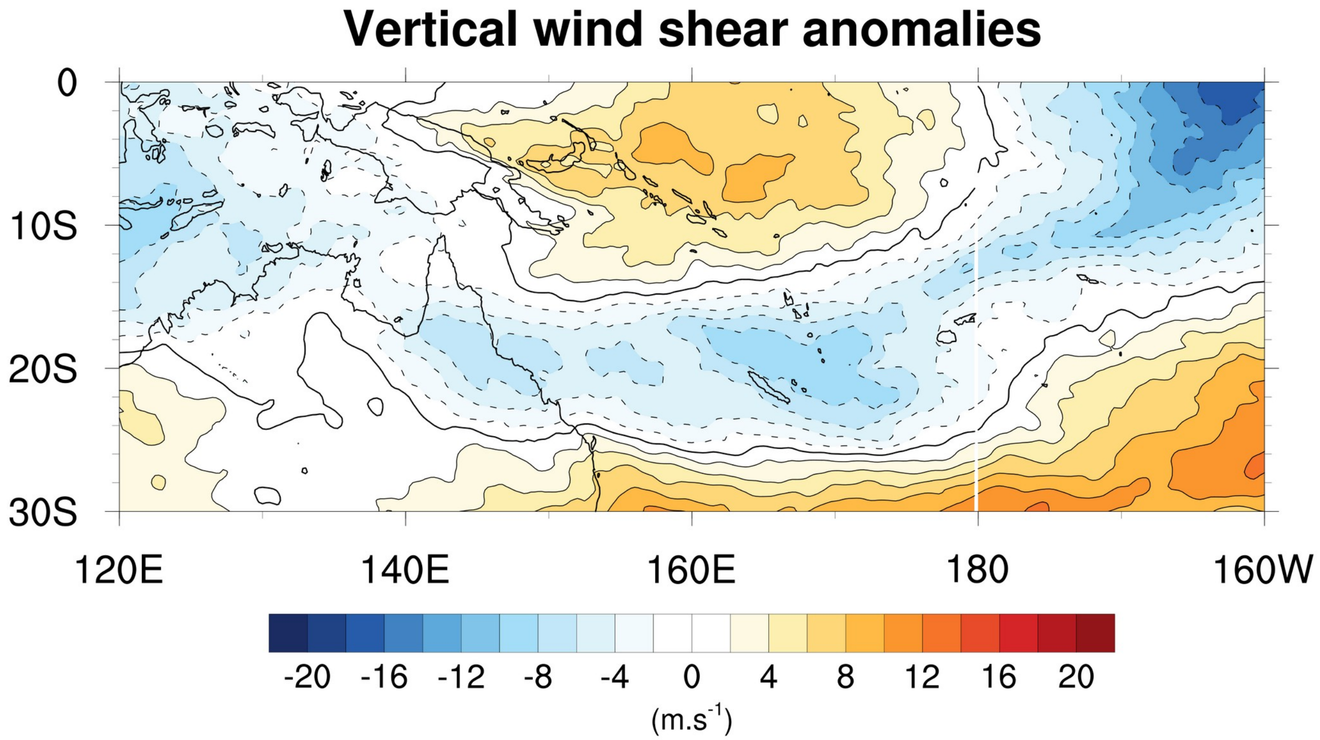

Fig. S9: Vertical wind shear anomalies (m.s^{-1}) in February 2016 from ERA5 reanalysis. Vertical wind shear is calculated as the wind speed difference between 200hPa and 850hPa.

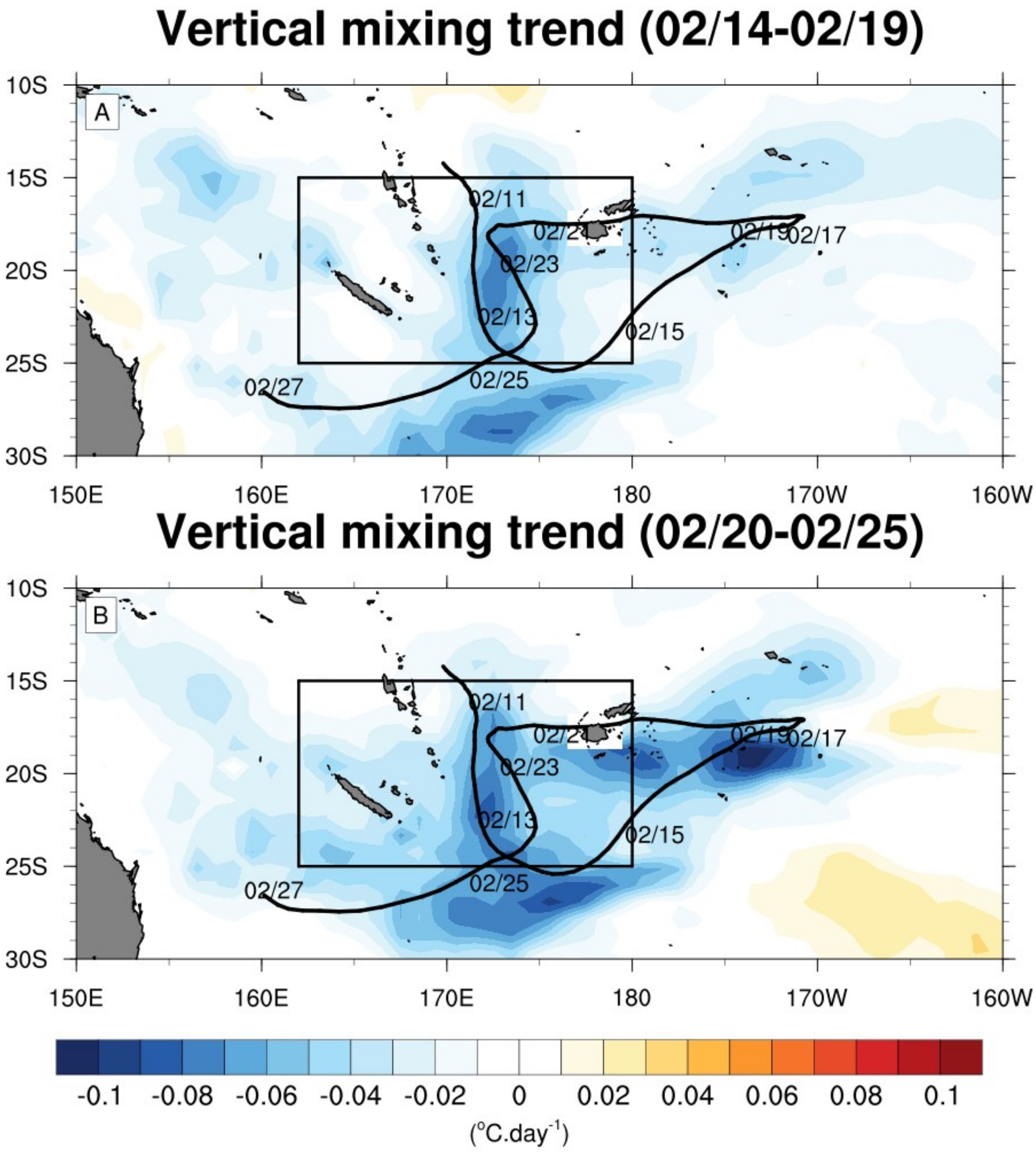

Fig. S10: The time-averaged between **(a)** 14th and 19th February and **(b)** 20th and 25th February of the vertical mixing term (°C.day^{-1}) from a NEMO simulation with online mixed layer temperature budget calculation.

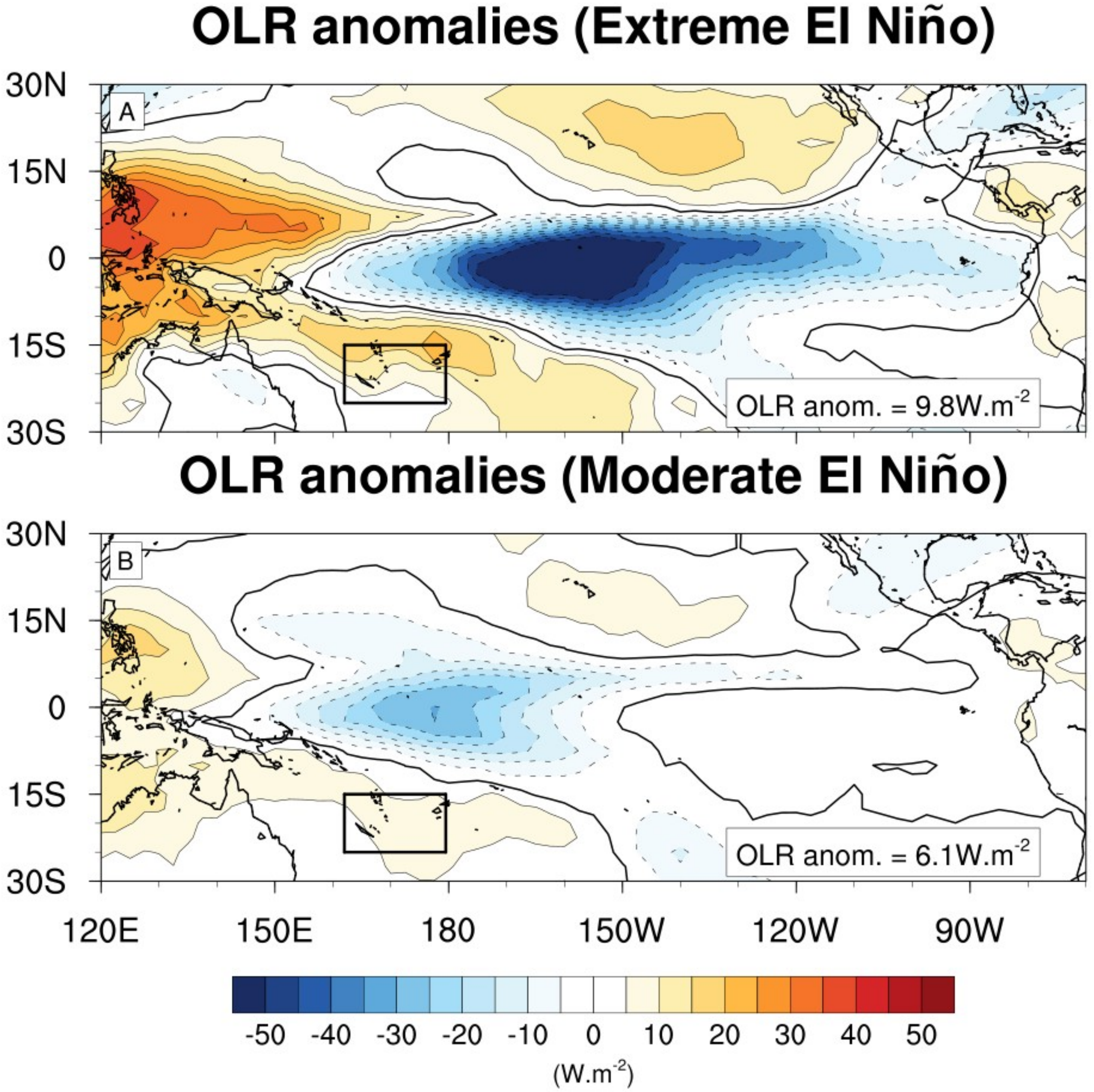

Fig. S11 : Composites of austral summer Outgoing Longwave Radiation (OLR) anomalies (in W.m^{-2}) during **(A)** Extreme El Niño and **(B)** Moderate El Niño events. The black box shows the study area [162°E-180°E; 15°S-25°S] used for the marine heatwave statistics and the temperature budget calculation. The spatial average of the OLR anomalies calculated in the study region is indicated on the bottom-right corner.

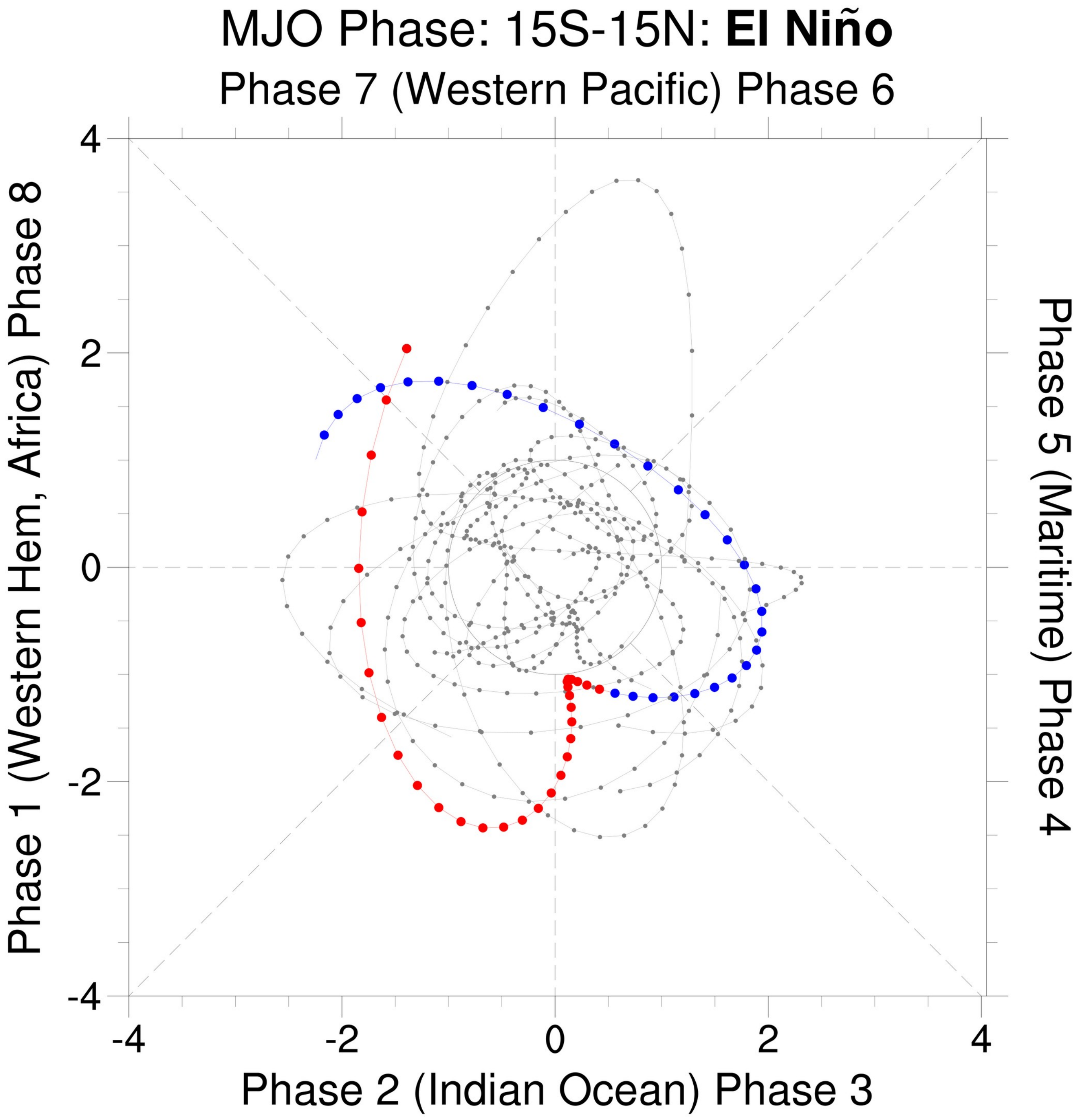

Fig. S12: Madden Julian Oscillation index from January to February for all El Niño years between 1993 and 2019. 2016 January days are in red, 2016 February days are in blue. The others El Niño years are in grey. The MJO index is calculated from ERA5 reanalysis.

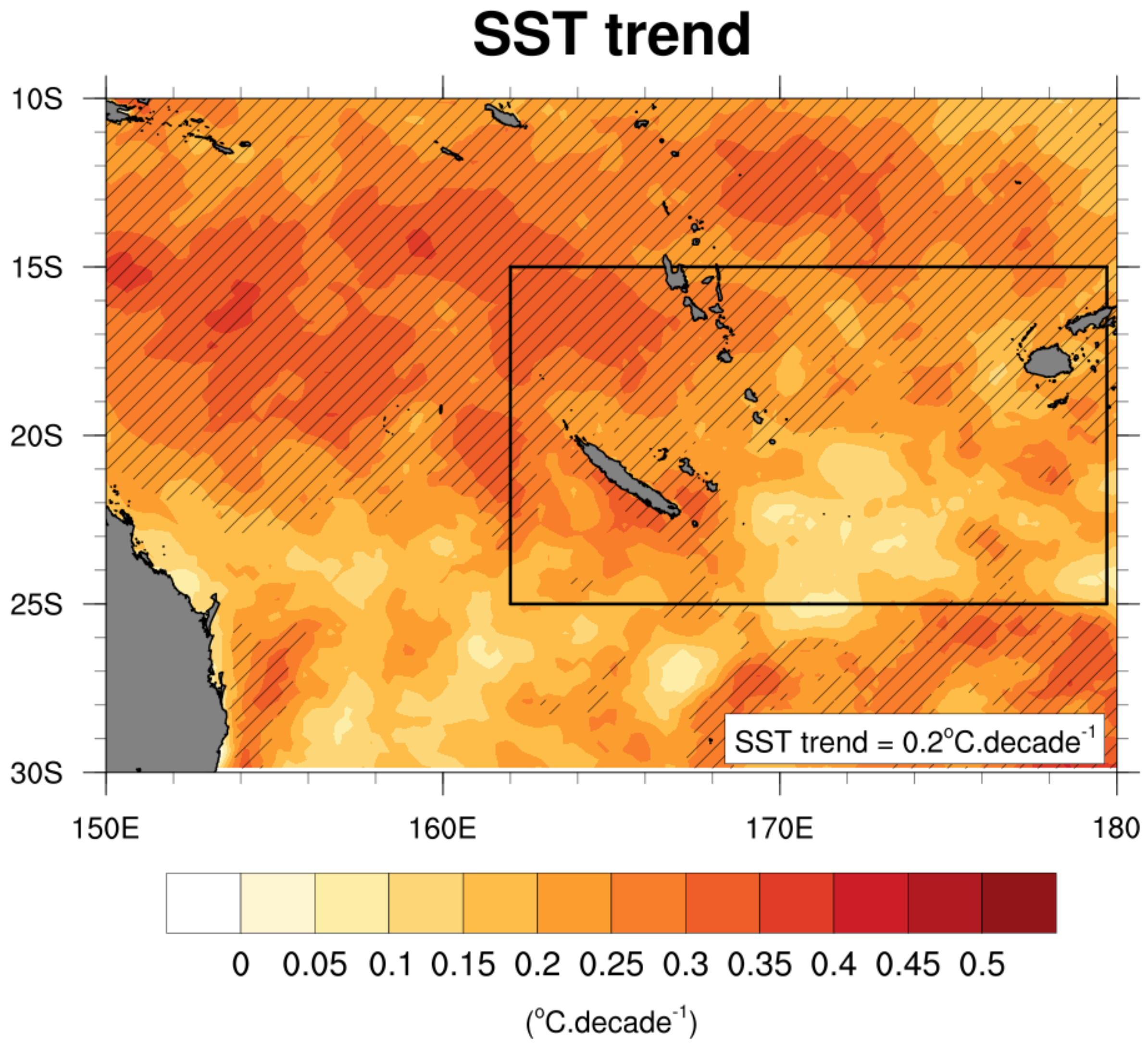

Fig. S13: Austral summer (December-Janaury-Febraury) sea surface temperature (SST) linear trend calculated over the 1993-2022 period from NOAA OI SST V2 High Resolution Dataset. The hatched areas represent the regions where the trends are significant at a threshold of 95% from a Mann–Kendall non-parametric test. The spatial average of the SST trend calculated in the study region is indicated on the bottom-right corner. Black box shows the region where MHW statistics and SST trend were calculated.

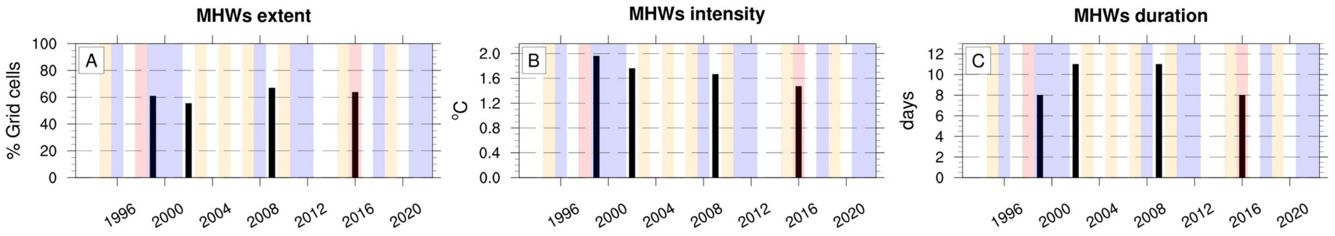

Fig. S14: Austral summer (December-Janaury-Febraury) marine heatwaves (MHWs) statistics from 1993 to 2022 for detrended sea surface temperature. **(A)** The maximum proportion of our study area experiencing MHW conditions (in % of grid cells), **(B)** maximum intensity (in °C) and **(C)** the duration of MHWs (in days). A MHW is detected when the event covers 50 % of our study region during at least 5 days.

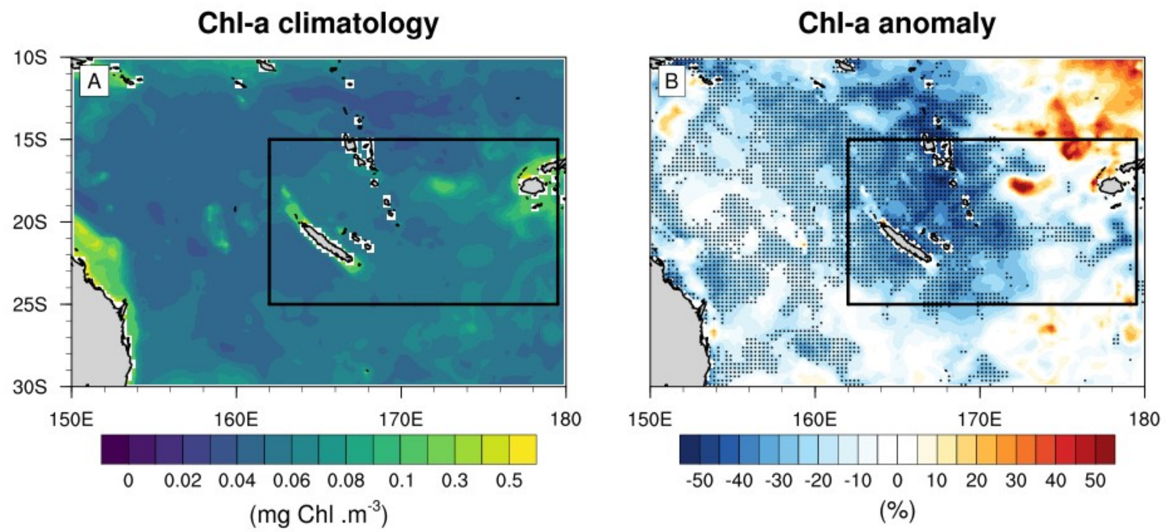

Fig. S15. 2016 marine heatwave (MHW) impacts on biology. **(A)** Climatology (in mg Chl . m^{-3}) and **(B)** anomalies (in %) of Chlorophyll-a concentration during the 2016 marine heatwaves. Regions where Chlorophyll-a concentrations during the 2016 marine heatwave are below the 10th percentile of the Chlorophyll-a concentration distribution are stippled. The black box shows the study area [162°E-180°E; 15°S-25°S] used for the MHW statistics and the temperature budget calculation.
